# Mitral Valve Prolapse and Anxiety Disorders: A Systematic Review of Evidence in Both Directions and Meta-Analysis

**DOI:** 10.3390/jcm15176792

**Published:** 2026-09-01

**Authors:** Andrea Sonaglioni, Giulio Francesco Gramaglia, Gian Luigi Nicolosi, Massimo Baravelli, Michele Lombardo

**Affiliations:** 1 Division of Cardiology, IRCCS MultiMedica, 20123 Milan, Italy; massimo.baravelli@multimedica.it (M.B.); michele.lombardo@multimedica.it (M.L.); 2 Department of Emergency, Fondazione IRCSS Ca’ Granda, Ospedale Maggiore Policlinico, 20122 Milan, Italy; giulio.gramaglia@unimi.it; 3 Division of Cardiology, Policlinico San Giorgio, 33170 Pordenone, Italy; gianluigi.nicolosi@gmail.com

**Keywords:** mitral valve prolapse, anxiety disorders, panic disorder, echocardiography, thoracic skeletal abnormalities, pectus excavatum, modified Haller index, systematic review, meta-analysis

## Abstract

**Background**: The association between mitral valve prolapse (MVP) and anxiety disorders has remained controversial for more than four decades because of heterogeneous psychiatric definitions, evolving echocardiographic diagnostic criteria, and conflicting findings across observational studies. We performed a systematic review to examine the available evidence in both directions of the MVP–anxiety relationship, together with a meta-analysis of anxiety-related outcomes among patients with MVP versus controls, and to evaluate the influence of different echocardiographic diagnostic criteria on this association. **Methods**: PubMed, Scopus, and EMBASE were systematically searched for observational studies evaluating either direction of the association between MVP and anxiety disorders. Studies assessing anxiety-related outcomes in patients with MVP or the prevalence of MVP among individuals with anxiety disorders were included in the systematic review. Quantitative synthesis was restricted to studies providing comparative data on dichotomous anxiety-related outcomes in MVP and control populations, using random-effects models to calculate pooled odds ratios (ORs) with 95% confidence intervals (CIs), whereas evidence concerning MVP prevalence among individuals with anxiety disorders was synthesized descriptively. **Results**: Seventeen studies published between 1979 and 2026 met the inclusion criteria, including 10 evaluating anxiety-related outcomes in patients with MVP and 7 assessing MVP prevalence among patients with anxiety disorders. Eight studies evaluating anxiety-related outcomes in MVP versus control populations were eligible for quantitative synthesis, whereas studies examining MVP among individuals with anxiety disorders contributed to the descriptive synthesis. Overall, patients with MVP showed a significantly higher prevalence of anxiety-related outcomes than controls (OR 1.78, 95% CI 1.15–2.75; *p* = 0.010), with no evidence of between-study heterogeneity (I^2^ = 0%). The association was significant in studies using 2D echocardiographic assessment but not in those using historical M-mode criteria, with no significant between-subgroup difference (*p* = 0.227). Meta-regression identified no significant influence of publication year, age, or sex on the pooled estimate. Sensitivity analyses showed that the pooled association was not disproportionately influenced by any single study. **Conclusions**: Patients with MVP exhibit a significantly increased prevalence of anxiety-related outcomes compared with controls, although no causal relationship or effect modification by echocardiographic method can be established. Conversely, evidence on MVP prevalence among individuals with anxiety disorders remains heterogeneous and currently supports only descriptive synthesis. Future prospective studies integrating standardized psychiatric assessment, contemporary echocardiographic criteria, and quantitative thoracic morphometry are warranted to clarify the underlying mechanisms and clinical implications.

## 1. Introduction

Mitral valve prolapse (MVP) is one of the most common valvular abnormalities, affecting approximately 2–3% of the general population [1,2,3] and representing the leading cause of primary mitral regurgitation in developed countries [4]. According to current echocardiographic criteria, MVP is defined as superior systolic displacement of one or both mitral valve leaflets by at least 2 mm beyond the mitral annular plane in the parasternal long-axis view, with or without associated leaflet thickening and mitral regurgitation [5,6]. Although many affected individuals remain asymptomatic throughout life, others experience a broad spectrum of symptoms, including palpitations, atypical chest pain, dyspnea, fatigue, orthostatic intolerance, and autonomic dysfunction, that are frequently disproportionate to the severity of valvular abnormalities [7,8,9]. This heterogeneous clinical presentation has long stimulated interest in the potential interaction between MVP and psychological disorders.

Among psychiatric conditions, anxiety disorders have received particular attention since the late 1970s, when the concept of the “mitral valve prolapse syndrome” was introduced to describe patients presenting with autonomic symptoms, panic attacks, chest discomfort, and palpitations in association with echocardiographic evidence of MVP [10]. Subsequent investigations reported an apparently increased prevalence of panic disorder and other anxiety disorders among patients with MVP, while several studies also suggested a higher frequency of MVP in individuals referred for psychiatric evaluation because of panic disorder [11]. These observations generated considerable interest regarding the existence of a shared pathophysiological substrate involving autonomic nervous system dysregulation, heightened adrenergic activity, connective tissue abnormalities, or altered perception of cardiac sensations [12,13].

Despite decades of research, the relationship between MVP and anxiety disorders remains controversial. Early studies were generally based on small clinical cohorts and relied on M-mode echocardiographic criteria [14,15,16], which substantially overestimated the prevalence of MVP compared with the more specific 2D echocardiographic definitions subsequently established and incorporated into contemporary recommendations [5,6]. In addition, considerable heterogeneity exists across the literature regarding patient selection, psychiatric diagnostic criteria, control populations, and the spectrum of anxiety disorders investigated. While several reports described a significantly higher prevalence of panic disorder or anxiety symptoms among patients with MVP, others failed to demonstrate any meaningful association after controlling for mitral regurgitation severity, symptom burden, or contemporary diagnostic criteria. More recent investigations have further suggested that psychological distress may be more closely related to patients’ subjective perception of illness or to symptomatic MVP rather than to the anatomical diagnosis of prolapse itself. Conversely, pediatric studies performed using current echocardiographic criteria have generally reported no significant increase in anxiety disorders compared with healthy controls, although symptomatic children with MVP often exhibit higher anxiety scores than asymptomatic patients [17,18].

These inconsistencies have important clinical implications. Anxiety symptoms may substantially impair quality of life, increase healthcare utilization, and complicate the diagnostic evaluation of patients presenting with chest pain, palpitations, or dyspnea. Conversely, attributing psychological symptoms solely to MVP without robust supporting evidence may contribute to unnecessary investigations, inappropriate reassurance strategies, or overdiagnosis of both cardiac and psychiatric conditions. Therefore, clarification of the true magnitude of the association between MVP and anxiety disorders remains clinically relevant for both cardiologists and mental health professionals.

Although numerous observational studies have examined this topic over the past four decades, no comprehensive systematic review with quantitative synthesis has specifically evaluated anxiety-related outcomes among patients with MVP compared with control populations while accounting for the evolution of echocardiographic diagnostic criteria. Accordingly, the present systematic review was undertaken to map and critically synthesize the available evidence in both directions of the MVP–anxiety relationship. The quantitative meta-analysis specifically evaluated study-defined dichotomous anxiety-related outcomes among patients with MVP compared with control populations, recognizing the heterogeneity of psychiatric constructs and assessment methods across the available literature. Evidence regarding the prevalence of MVP among individuals with anxiety disorders was synthesized descriptively because the available studies did not permit a sufficiently homogeneous comparative quantitative analysis. In addition to pooling comparative data from eligible case–control and cohort studies, we performed a comprehensive descriptive analysis of study design, patient characteristics, psychiatric assessment methods, and echocardiographic diagnostic criteria to provide an integrated overview of the current evidence and identify potential sources of methodological and clinical heterogeneity across the literature.

## 2. Materials and Methods

This systematic review and meta-analysis was conducted in accordance with the Preferred Reporting Items for Systematic Reviews and Meta-Analyses (PRISMA) 2020 Statement [19]. The completed PRISMA 2020 checklist is provided in the Supplementary Materials (Supplementary Materials File S1).

The review process was initiated on 5 July 2026 with the literature search. Study screening was conducted from 10 July to 17 July 2026, data extraction from 18 July to 21 July 2026, and statistical analyses from 22 July to 31 July 2026. The methodological protocol had been finalized before initiation of the literature search, and no amendments to the predefined eligibility criteria, data extraction framework, or principal analytical strategy were made during the review process. The study protocol was subsequently registered in the International Platform of Registered Systematic Review and Meta-analysis Protocols (INPLASY) on 1 August 2026 (registration number INPLASY202680006). The complete INPLASY record is provided in the Supplementary Materials (Supplementary Materials File S2).

### 2.1. Literature Search Strategy

A comprehensive literature search was independently performed by two investigators to identify studies evaluating the relationship between MVP and anxiety disorders in either direction. Three electronic databases (PubMed, Scopus, and EMBASE) were systematically searched from database inception through 9 July 2026, without restrictions regarding publication year, language, or country of origin. No language restriction was applied during study screening or eligibility assessment; however, all studies ultimately meeting the inclusion criteria were available in English, and therefore no translation of included reports was required.

The search strategy combined terms related to MVP and psychiatric outcomes. The following prespecified search combinations were applied across the three databases: “mitral valve prolapse” AND “anxiety disorders”; “mitral valve prolapse” AND “anxiety” AND “prevalence”; “mitral valve prolapse” AND “panic disorder”; “mitral valve prolapse” AND “agoraphobia”; and “mitral valve prolapse” AND “depression”. The term “depression” was included as a sensitivity-enhancing search term to identify studies evaluating broader or overlapping psychiatric phenotypes in which anxiety-related outcomes might also be reported; depression itself was not considered an eligible anxiety-related outcome. The complete database-specific search strategies and date of the final search are provided in Supplementary Materials File S3.

Because the review addressed two complementary research questions, studies investigating the prevalence of anxiety disorders among patients with MVP and those evaluating the prevalence of MVP among individuals with anxiety disorders were identified through the same search strategy and screened simultaneously. The reference lists of all eligible studies and relevant review articles were also manually searched to identify additional publications not retrieved through the electronic database search.

All retrieved citations were imported into a reference management software, and duplicate records were removed before screening. Titles and abstracts were independently reviewed by two investigators, followed by full-text evaluation of potentially eligible studies. Any disagreements regarding study eligibility were resolved through discussion and consensus.

### 2.2. Study Eligibility Criteria

Studies were considered eligible if they were original observational investigations, including prospective or retrospective cohort studies, case–control studies, cross-sectional studies, or population-based database analyses, evaluating the association between MVP and anxiety disorders. Specifically, studies were included if they investigated either the prevalence of anxiety disorders among individuals with MVP or the prevalence of MVP among patients with anxiety disorders.

Given that the review addressed two complementary research questions, studies were classified into two predefined analytical groups. The first comprised investigations comparing the frequency or severity of anxiety disorders between patients with MVP and control subjects. The second included studies evaluating the prevalence of MVP in cohorts with anxiety disorders relative to control populations or established reference values. Only studies including an appropriate comparison group were eligible for quantitative synthesis, whereas investigations lacking a control group were retained for the qualitative systematic review and descriptive analyses but were excluded from pooled effect-size calculations.

MVP diagnosis had to be established by echocardiography using the diagnostic criteria adopted by the original investigators. Both historical M-mode definitions [14,15,16] and 2D echocardiographic assessment using the study-specific diagnostic definitions, including contemporary guideline-based criteria where applicable [5,6], were considered eligible because one objective of the review was to evaluate the potential influence of evolving diagnostic approaches on the reported association between MVP and anxiety disorders. Similarly, anxiety disorders could be identified using standardized psychiatric interviews, validated psychometric questionnaires, or internationally recognized diagnostic classification systems (e.g., DSM-based criteria or ICD codes), provided that the diagnostic methodology was clearly described.

No restrictions were imposed regarding participant age, sex, geographical region, or clinical setting. Pediatric studies were therefore considered eligible when they fulfilled the predefined inclusion criteria. Studies involving mixed populations were included only when data relating to participants with MVP or anxiety disorders could be extracted separately.

Editorials, narrative reviews, systematic reviews, meta-analyses, conference abstracts, case reports, letters, expert opinions, and clinical practice guidelines were excluded. Studies lacking sufficient quantitative information to determine the prevalence of MVP or anxiety disorders, calculate odds ratios, or extract the necessary study-level data were also excluded from the quantitative meta-analysis.

### 2.3. Data Extraction and Study Classification

Two reviewers independently performed study screening and selection. After duplicate citations had been removed, titles and abstracts were examined to identify potentially relevant reports. Articles considered eligible at this stage were retrieved in full text and evaluated against the prespecified selection criteria. Differences in judgment were resolved through discussion; a third investigator adjudicated when agreement could not be reached. Formal inter-reviewer agreement statistics were not calculated because independent pre-consensus screening decisions were not prospectively retained for this purpose.

For each publication, the following information was recorded: first author, country, publication year, study design, recruitment setting, population characteristics, sample size, psychiatric assessment method, echocardiographic or administrative criteria used to identify MVP, and principal study findings. The included investigations were then assigned to one of two predefined groups according to whether they assessed anxiety outcomes among participants with MVP or the prevalence of MVP among individuals with anxiety disorders.

For studies examining anxiety among patients with MVP, extracted demographic and clinical variables included age, sex, body mass index, systolic blood pressure, resting or standing heart rate, left ventricular ejection fraction, and systolic pulmonary artery pressure, whenever reported. Psychiatric information included the type of anxiety or panic disorder, the number of affected participants, and scores obtained using the instruments employed in the original studies. To distinguish clinically diagnosed psychiatric disorders from symptom-based constructs, anxiety-related outcomes were classified according to the ascertainment method used in the original study. Clinically diagnosed anxiety or panic disorders were defined as outcomes established through structured psychiatric assessment or recognized diagnostic criteria, whereas anxiety symptom burden comprised outcomes assessed using validated psychometric instruments without requiring a formal psychiatric diagnosis. Diagnostic assessment was based on structured interviews, including the Diagnostic and Statistical Manual of Mental Disorders (DSM)-based diagnostic interviews, the Structured Clinical Interview for DSM Disorders (SCID), the Mini International Neuropsychiatric Interview (MINI), and the Diagnostic Interview Schedule (DIS). Symptom severity was assessed using validated psychometric instruments, including the Zung Self-Rating Anxiety Scale (SAS), the anxiety domain of the Symptom Checklist-90-Revised (SCL-90-R), the Hospital Anxiety and Depression Scale–Anxiety subscale (HADS-A), the State–Trait Anxiety Inventory (STAI), the State–Trait Cardiac Anxiety Scale (STCAS), the Screen for Child Anxiety Related Emotional Disorders (SCARED), the DSM-5 Separation Anxiety Disorder Severity Scale, the Somatosensory Amplification Scale (SSAS), and parental overprotection or anxiety-sensitivity instruments. Because reporting differed markedly across studies, the number of participants contributing to each descriptive variable was recorded separately.

For investigations evaluating MVP among patients with anxiety or panic disorders, the variables collected included total cohort size, age, sex distribution, number and proportion of participants diagnosed with MVP, age and sex characteristics of the MVP subgroup, psychiatric diagnosis, psychiatric diagnostic framework, and the cardiac method used to establish MVP. When available, additional electrocardiographic, exercise-related, or echocardiographic measurements were also extracted. The overall number of anxiety-disorder participants, the number diagnosed with MVP, and study-level MVP prevalence estimates were used for the descriptive synthesis.

MVP ascertainment was documented exactly as reported by each study. Historical investigations commonly used M-mode echocardiographic definitions based on posterior systolic leaflet displacement, whereas later studies applied 2D criteria based on superior systolic displacement of one or both mitral leaflets beyond the annular plane. Studies identifying mitral valve disorders through diagnostic codes were retained for qualitative synthesis, but the less specific nature of such definitions was considered during methodological appraisal.

Given the heterogeneity of psychiatric assessment across studies, outcome harmonization for quantitative synthesis was based exclusively on dichotomous anxiety-related outcomes explicitly reported by the original investigators. These dichotomous outcomes were further classified as either (i) clinically diagnosed anxiety or panic disorders established using structured psychiatric assessment or recognized diagnostic criteria, or (ii) instrument-defined anxiety-related outcomes derived from validated psychometric measures using thresholds specified by the original investigators. This distinction was introduced to avoid treating formal psychiatric diagnoses and symptom-based psychological constructs as clinically equivalent endpoints. Continuous psychometric scores were analyzed descriptively and were not transformed into categorical outcomes unless the original study provided a predefined diagnostic or categorical threshold. Accordingly, continuous measures of anxiety severity, cardiac anxiety, anxiety sensitivity, somatosensory amplification, and related psychological constructs did not contribute directly to the pooled dichotomous effect estimate.

For the comparative meta-analysis, the numbers of participants with and without the study-specific dichotomous anxiety-related outcome in the MVP and control groups were extracted to construct study-specific 2 × 2 contingency tables and calculate odds ratios (ORs) with 95% confidence intervals (CIs). The overall analysis therefore represents a synthesis of study-defined dichotomous anxiety-related outcomes rather than a pooled prevalence estimate of a single, uniformly defined psychiatric disorder. Where the available event and denominator data permitted, mutually independent datasets with sufficiently detailed information were additionally analyzed separately according to clinically diagnosed anxiety/panic disorders versus instrument-defined anxiety-related outcomes to assess whether the observed association was consistent across these clinically distinct outcome categories. Potential cohort overlap was assessed by comparing author lists, recruitment centers and settings, study periods, participant characteristics, sample sizes, and descriptions of the source populations across eligible reports. When reports originated from potentially overlapping populations, their recruitment characteristics and study samples were cross-checked to avoid duplicate inclusion of the same participants. No potentially overlapping datasets were identified among the studies included in the quantitative meta-analysis.

Weighted descriptive summaries were derived from aggregate study-level data after completion of data extraction. Each extracted value was independently cross-checked against the original publication by the second reviewer. Any discrepancies were resolved through joint review of the source article until consensus was reached.

### 2.4. Statistical Analysis

Aggregate study-level data were first summarized descriptively to characterize the two complementary populations examined in this review. For studies evaluating anxiety-related outcomes among individuals with MVP, continuous variables were expressed as sample size-weighted mean values and categorical variables as weighted proportions. The number of participants contributing to each estimate was reported because data availability differed across variables. The same approach was applied to cohorts of patients with anxiety or panic disorders evaluated for MVP. These weighted summaries were used exclusively for descriptive characterization of the available study populations; no inferential between-group testing was performed because the contributing studies and sample sizes differed across variables and individual patient-level data were unavailable.

The quantitative synthesis was restricted to studies providing sufficient event and denominator data for both MVP and control groups. For each investigation, the association between MVP and the study-specific dichotomous anxiety-related outcome was expressed as an OR with its corresponding 95% CI. For studies containing a single zero-event cell, a continuity correction of 0.5 was added to all four cells of the corresponding 2 × 2 table before calculation of the log OR and its variance. Random-effects pooling was performed using the DerSimonian–Laird method-of-moments estimator for between-study variance (τ^2^), with inverse-variance weighting. Conventional 95% CIs were calculated on the log-OR scale using the normal approximation and subsequently exponentiated. Because clinically relevant differences were anticipated in recruitment setting, psychiatric assessment, participant age, and echocardiographic definition of MVP, a random-effects framework was selected a priori. Fixed-effect estimates were additionally examined as a consistency analysis; when the estimated between-study variance was zero, the two approaches yielded identical pooled results.

Statistical heterogeneity was assessed using Cochran’s Q test and quantified with the I^2^ statistic. I^2^ values of approximately 25%, 50%, and 75% were conventionally interpreted as indicating low, moderate, and substantial statistical heterogeneity, respectively, while between-study variance was represented by τ^2^. Given the limited number of studies, a modified Hartung–Knapp adjustment was additionally applied as a conservative sensitivity analysis. A 95% prediction interval was calculated on the log-OR scale incorporating the estimated between-study variance and subsequently back-transformed to the OR scale to estimate the range of effects that might be expected in a comparable future study. A two-sided *p* value below 0.05 was considered statistically significant.

A prespecified subgroup analysis was conducted according to the echocardiographic method used to diagnose MVP. Studies relying on historical M-mode assessment were analyzed separately from those using 2D echocardiographic assessment, irrespective of the specific study-level 2D diagnostic definition. Differences between subgroup estimates were formally evaluated using the between-group Q statistic.

Potential small-study effects were examined through visual inspection of funnel plot symmetry and formally assessed using Egger’s regression intercept. Given that fewer than 10 studies contributed to the meta-analysis, the results of funnel plot-based analyses were interpreted cautiously because of their limited statistical power.

To explore whether selected study-level characteristics might contribute to variability in the pooled association, an exploratory random-effects multivariable meta-regression model was fitted including publication year, mean age, and proportion of male participants as candidate moderators. Regression coefficients represented the estimated change in the log OR associated with each covariate after accounting for the other study-level moderators and were reported with standard errors, 95% CIs, and two-sided *p* values. Given the very small number of studies relative to the number of moderators, the resulting estimates were considered potentially unstable and the analysis was regarded as highly exploratory rather than confirmatory. Moreover, because only aggregate study-level data were available, the possibility of ecological bias could not be excluded.

The temporal stability of the pooled estimate was further explored through a cumulative meta-analysis, in which studies were added sequentially according to publication year. This analysis was used to determine how the magnitude, precision, and statistical significance of the association evolved as additional evidence became available.

Sensitivity analyses were performed using a leave-one-out approach, in which the pooled effect size was recalculated after sequential omission of each individual study. This procedure was used to evaluate the robustness of the overall estimate and to determine whether the pooled association was disproportionately influenced by any single investigation.

All meta-analytic procedures were performed using Comprehensive Meta-Analysis software, version 3.0 (Biostat, Englewood, NJ, USA).

### 2.5. Assessment of Methodological Quality and Risk of Bias

The methodological quality of all eligible studies was independently evaluated by two investigators using design-specific National Institutes of Health (NIH) Quality Assessment Tools [20]. Studies with a case–control design were assessed using the NIH Quality Assessment Tool for Case–Control Studies, whereas cohort and cross-sectional investigations were evaluated using the NIH Quality Assessment Tool for Observational Cohort and Cross-Sectional Studies. These standardized appraisal instruments examine key methodological features relevant to each study design, including the clarity of the research question, definition and selection of the study population, exposure and outcome ascertainment, reliability of measurements, blinding where applicable, control of potential confounding, and appropriateness of the analytical approach.

Individual methodological domains were rated as “Yes”, “No”, “Cannot Determine”, “Not Reported”, or “Not Applicable”, as appropriate. Consistent with the NIH approach, domain ratings were not summed to generate numerical quality scores or converted into predefined numerical thresholds. Instead, an overall qualitative judgment of Good, Fair, or Poor methodological quality was assigned after considering the pattern and relative importance of potential sources of bias across the applicable domains, with particular attention to participant selection, exposure and outcome ascertainment, temporal relationships, blinding, and control of potential confounding.

Any discrepancies in methodological quality assessment between the two reviewers were resolved through joint review of the original publication and discussion until consensus was achieved. Formal inter-reviewer agreement statistics were not calculated because independent pre-consensus ratings were not prospectively retained for this purpose.

### 2.6. Certainty of Evidence

The certainty of the evidence was additionally assessed using a GRADE-based approach adapted to observational evidence [21]. Because the included studies were predominantly observational, the initial certainty was considered low. The overall body of evidence was then evaluated across the principal GRADE domains of risk of bias, inconsistency, indirectness, imprecision, and publication bias. Potential upgrading factors, including a large magnitude of association, evidence of a dose–response relationship, and the possible influence of residual confounding on the observed effect, were also considered when applicable. Certainty was assessed separately for the principal review questions and categorized as high, moderate, low, or very low. The final judgment reflected the overall strength and limitations of the available evidence rather than the statistical significance of individual studies or pooled estimates.

### 2.7. Statement on the Use of Artificial Intelligence

Artificial intelligence-based software was used exclusively to improve the linguistic quality, readability, and overall clarity of the manuscript. Specifically, ChatGPT (OpenAI, San Francisco, CA, USA; GPT-5.5) was employed to assist with grammar correction, stylistic refinement, sentence restructuring, and language editing.

Artificial intelligence was not involved in the conception or design of the study, literature searching, study selection, data extraction, methodological quality assessment, statistical analyses, interpretation of the results, or formulation of the scientific conclusions. All methodological decisions, analytical procedures, interpretation of findings, and the final scientific content were independently conceived, critically reviewed, and approved by the authors, who assume full responsibility for the accuracy, integrity, and originality of the work.

## 3. Results

### 3.1. Study Identification and Selection Process

The database search yielded a total of 510 records, including 185 from PubMed, 165 from Scopus, and 160 from EMBASE. Following the removal of 350 duplicate citations, 160 unique articles underwent title and abstract screening. Based on the predefined eligibility criteria, 115 records were excluded at this stage.

The full texts of the remaining 45 articles were sought for retrieval. Full-text retrieval was attempted through all three electronic databases used for the systematic search (PubMed, Scopus, and EMBASE); however, despite these attempts, 10 reports could not be retrieved and were therefore not available for formal eligibility assessment. These records had been retained after title and abstract screening because the available bibliographic information indicated potential relevance to the relationship between MVP and anxiety disorders; however, their eligibility could not be confirmed without assessment of the complete reports. Consequently, 35 full-text articles were assessed for eligibility. The bibliographic details of the 10 unavailable reports are provided in Supplementary Materials File S4 to ensure transparency regarding potentially relevant evidence that could not undergo full-text assessment. Among the 35 retrieved articles, 18 were subsequently excluded, including 9 because they did not provide sufficient information regarding anxiety disorders and 9 because the diagnosis of MVP could not be reliably confirmed according to the predefined inclusion criteria.

A total of 17 studies met the inclusion criteria and were included in the qualitative systematic review. Of these, 10 [22,23,24,25,26,27,28,29,30,31] investigated the prevalence of anxiety disorders among individuals with MVP, whereas 7 [32,33,34,35,36,37,38] evaluated the prevalence of MVP among patients with anxiety disorders. Eight studies reporting appropriate control groups [22,23,24,25,26,28,30,31] were eligible for quantitative synthesis and were therefore included in the meta-analysis assessing anxiety-related outcomes in patients with MVP.

**Figure 1:**
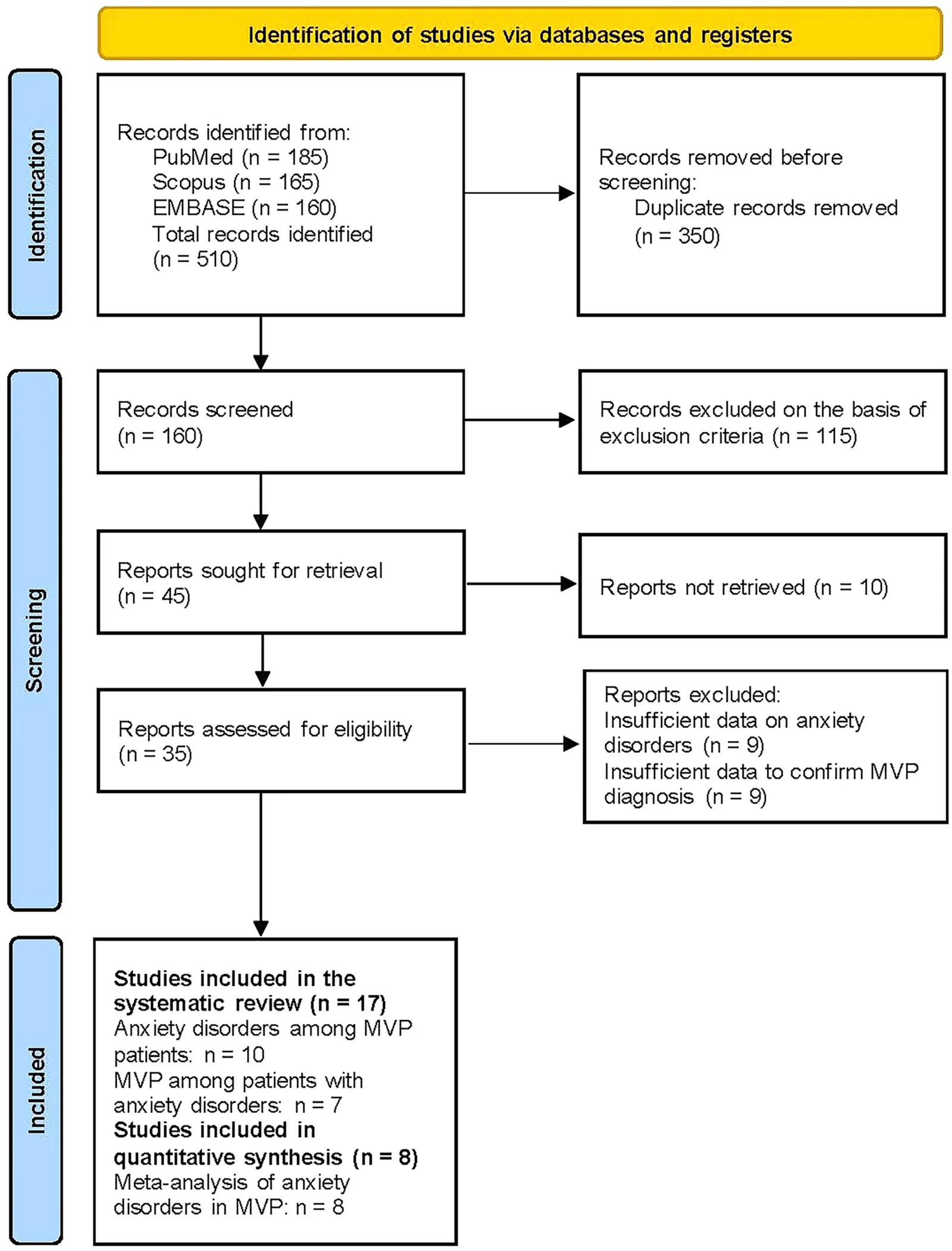
PRISMA 2020 flow diagram illustrating the study selection process, from database identification and screening to eligibility assessment and final inclusion in the systematic review and meta-analysis.

The complete study selection process is illustrated in Figure 1 and is reported according to the PRISMA 2020 recommendations.

### 3.2. Study Characteristics and Methodological Overview

The 17 studies included in the systematic review were published between 1979 and 2026 and explored the relationship between MVP and anxiety disorders from two complementary perspectives. Specifically, 10 studies investigated the prevalence of anxiety disorders or anxiety-related symptoms among individuals with MVP, whereas 7 studies evaluated the prevalence of MVP in patients with anxiety disorders.

Overall, the available evidence was derived predominantly from prospective single-center case–control, cross-sectional, and observational studies conducted across North America, Europe, the Middle East, and Asia, although one nationwide population-based database study and one cohort study were also included. Substantial methodological heterogeneity was observed across studies with regard to participant selection, psychiatric diagnostic criteria, echocardiographic assessment, and outcome definitions. Earlier investigations [22,23,24] primarily relied on M-mode echocardiography for MVP diagnosis, whereas more recent studies [25,26,28,29,30,31] predominantly adopted 2D echocardiographic assessment. However, the specific 2D diagnostic definitions were not uniform across studies and did not necessarily correspond to the current criterion of systolic leaflet displacement ≥2 mm beyond the mitral annular plane in the parasternal long-axis view. Likewise, anxiety disorders were identified using a broad range of structured diagnostic interviews and validated psychometric instruments, including DSM-based interviews, SCID, MINI, DIS, HADS, STAI, SCARED, and other standardized anxiety questionnaires.

The methodological characteristics, study populations, psychiatric assessment methods, MVP diagnostic criteria, and principal findings of studies evaluating anxiety disorders among patients with MVP are summarized in Table 1, whereas the corresponding characteristics of studies investigating the prevalence of MVP among patients with anxiety disorders are presented separately in Table 2.

Overall, the available evidence illustrates the transition from early small clinical studies focused mainly on panic disorder to more recent investigations encompassing broader anxiety phenotypes, pediatric populations, and large-scale epidemiological cohorts, although substantial heterogeneity in methodology and diagnostic approaches persists across studies.

Despite these methodological differences, the principal findings showed a heterogeneous pattern. Among studies evaluating anxiety-related outcomes in patients with MVP, several investigations reported a higher prevalence of panic disorder, separation anxiety, or increased anxiety symptom scores, whereas others found no significant association compared with control populations or suggested that anxiety was primarily confined to symptomatic MVP subgroups. Conversely, studies investigating the prevalence of MVP among patients with anxiety disorders produced inconsistent results, with some early reports describing a markedly increased prevalence of MVP in panic disorder, while later investigations using more stringent echocardiographic criteria generally observed prevalence rates comparable to those of the general population and failed to confirm a consistent association.

### 3.3. Clinical and Psychiatric Profile of Patients with Mitral Valve Prolapse

The pooled demographic, clinical, and psychiatric characteristics of patients with MVP and control subjects are summarized in Table 3.

The included populations were predominantly young adults, with sample size-weighted mean ages of 28.4 years among patients with MVP and 29.5 years among controls. Available study-level data also provided descriptive information on body mass index, systolic blood pressure, resting heart rate, left ventricular ejection fraction, and systolic pulmonary artery pressure. However, the number and composition of participants contributing to individual variables differed substantially across studies and between groups; therefore, these aggregate estimates are intended solely to characterize the available study populations and should not be interpreted as formal between-group comparisons.

Considerable variability was observed in the assessment of anxiety, reflecting the use of multiple diagnostic interviews and psychometric instruments across studies. Descriptively, higher sample size-weighted scores among patients with MVP were observed for several anxiety-related measures, including the DSM-5 Separation Anxiety Disorder Severity Scale, Zung Self-Rating Anxiety Scale, Somatosensory Amplification Scale, and State–Trait Cardiac Anxiety Scale, whereas smaller differences were observed for SCARED, HADS-A, and SCL-90-R anxiety scores. These descriptive differences should be interpreted cautiously because each instrument was reported by a different subset of studies and participants and no formal pooled between-group comparison was performed for these continuous outcomes.

Across the available studies reporting categorical psychiatric outcomes, the descriptive aggregate prevalence of anxiety or panic disorders was 16.1% among patients with MVP and 11.3% among controls. These crude aggregate percentages were derived from different contributing study populations and are presented for descriptive purposes only; they should not be interpreted as an independent comparison or as confirmation of the primary meta-analytic estimate. The association between MVP and anxiety disorders is formally evaluated using within-study comparative data in the quantitative meta-analysis presented in Section 3.5.

### 3.4. Clinical Characteristics of Patients with Anxiety Disorders Screened for Mitral Valve Prolapse

The weighted demographic and clinical characteristics of patients with anxiety or panic disorders included in studies evaluating the prevalence of MVP are summarized in Table 4.

Overall, the pooled population comprised 672 patients with anxiety disorders, with a weighted mean age of 28.2 years, and women represented the majority of participants, accounting for approximately 60% of the study population.

Across the included studies, 89 of 672 patients were diagnosed with MVP, corresponding to a crude aggregate prevalence of 13.2%. This value represents the proportion obtained by summing MVP cases and participants across the contributing studies and is presented for descriptive purposes only; it should not be interpreted as a meta-analytic pooled prevalence estimate. Patients with both anxiety disorders and MVP were, on average, older than the overall anxiety cohorts (mean age 36.5 years) and remained predominantly female, with men accounting for 33.3% of MVP cases.

Although the pooled demographic characteristics were relatively homogeneous, the included studies differed substantially in psychiatric case definition, echocardiographic diagnostic criteria, and recruitment setting. In particular, earlier investigations focused primarily on patients with panic disorder recruited from specialized psychiatric clinics, whereas more recent studies also included broader anxiety phenotypes, such as social anxiety disorder and childhood anxiety disorders, and applied contemporary echocardiographic criteria for MVP diagnosis. These methodological differences should be considered when interpreting the reported prevalence estimates.

### 3.5. Quantitative Synthesis of the Association Between Mitral Valve Prolapse and Anxiety-Related Outcomes

Because the included studies assessed different anxiety-related constructs, outcome harmonization was based on the study-specific dichotomous psychiatric endpoint reported by the original investigators. Continuous psychometric scores were retained for descriptive analyses and were not dichotomized unless a categorical endpoint was explicitly reported. The exact psychiatric endpoint, assessment method, and event and denominator data used to construct each 2 × 2 contingency table are provided in Supplementary Materials File S5 [22,23,24,25,26,28,30,31].

**Figure 2:**
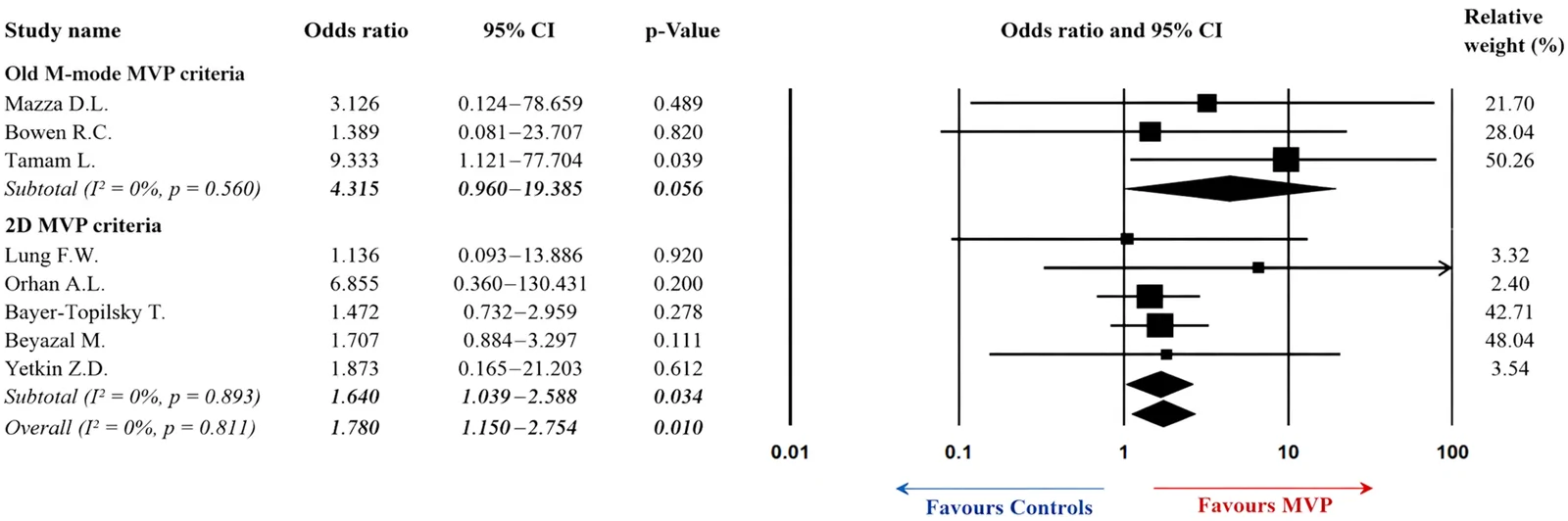
Forest plot of the meta-analysis comparing the prevalence of anxiety-related outcomes in patients with mitral valve prolapse (MVP) and control subjects. Studies were stratified according to the echocardiographic method used for MVP diagnosis into those based on historical M-mode criteria [22,23,24] and those using 2D echocardiographic assessment [25,26,28,30,31]. Diamonds represent the pooled odds ratios (ORs) with 95% confidence intervals (CIs) for each subgroup and for the overall analysis.

The pooled analysis of the eight studies [22,23,24,25,26,28,30,31] comparing anxiety-related outcomes between patients with MVP and control subjects is presented in Figure 2.

Overall, patients with MVP showed a significantly higher likelihood of anxiety-related outcomes than controls (OR 1.78, 95% CI 1.15–2.75, *p* = 0.010). No statistical between-study heterogeneity was detected (I^2^ = 0%, *p* = 0.811), although this finding should be interpreted in light of the limited number of studies included in the quantitative synthesis. Between-study variance was estimated as τ^2^ = 0.000. A modified Hartung–Knapp sensitivity analysis yielded the same pooled OR of 1.78 but a wider 95% CI of 1.05–3.01; the association remained statistically significant under this more conservative inferential approach. The corresponding 95% prediction interval was 1.03–3.07, although this estimate should be interpreted cautiously given the small number of studies and the absence of detectable between-study variance.

To explore the potential influence of evolving echocardiographic diagnostic criteria for MVP, a predefined subgroup analysis was performed according to the imaging modality used for diagnosis. Studies employing older M-mode echocardiographic criteria [22,23,24] showed a higher point estimate for anxiety-related outcomes among patients with MVP (OR 4.32, 95% CI 0.96–19.39), although the association did not reach statistical significance (*p* = 0.056). Studies using 2D echocardiographic assessment [25,26,28,30,31] demonstrated a significant association between MVP and anxiety-related outcomes (OR 1.64, 95% CI 1.04–2.59, *p* = 0.034). Importantly, the formal test for interaction showed no statistically significant difference between the M-mode and 2D subgroup estimates (Qbetween = 1.458, df = 1, *p* = 0.227).

Accordingly, although the point estimate was numerically higher among studies using historical M-mode criteria, the available data do not provide evidence that the association between MVP and anxiety-related outcomes differs according to echocardiographic diagnostic method. The considerably wider CI in the M-mode subgroup reflects the smaller sample size and lower precision of the earlier studies. The significant estimate observed in the 2D subgroup should therefore not be interpreted as evidence of a statistically significant difference from the M-mode subgroup.

**Figure 3:**
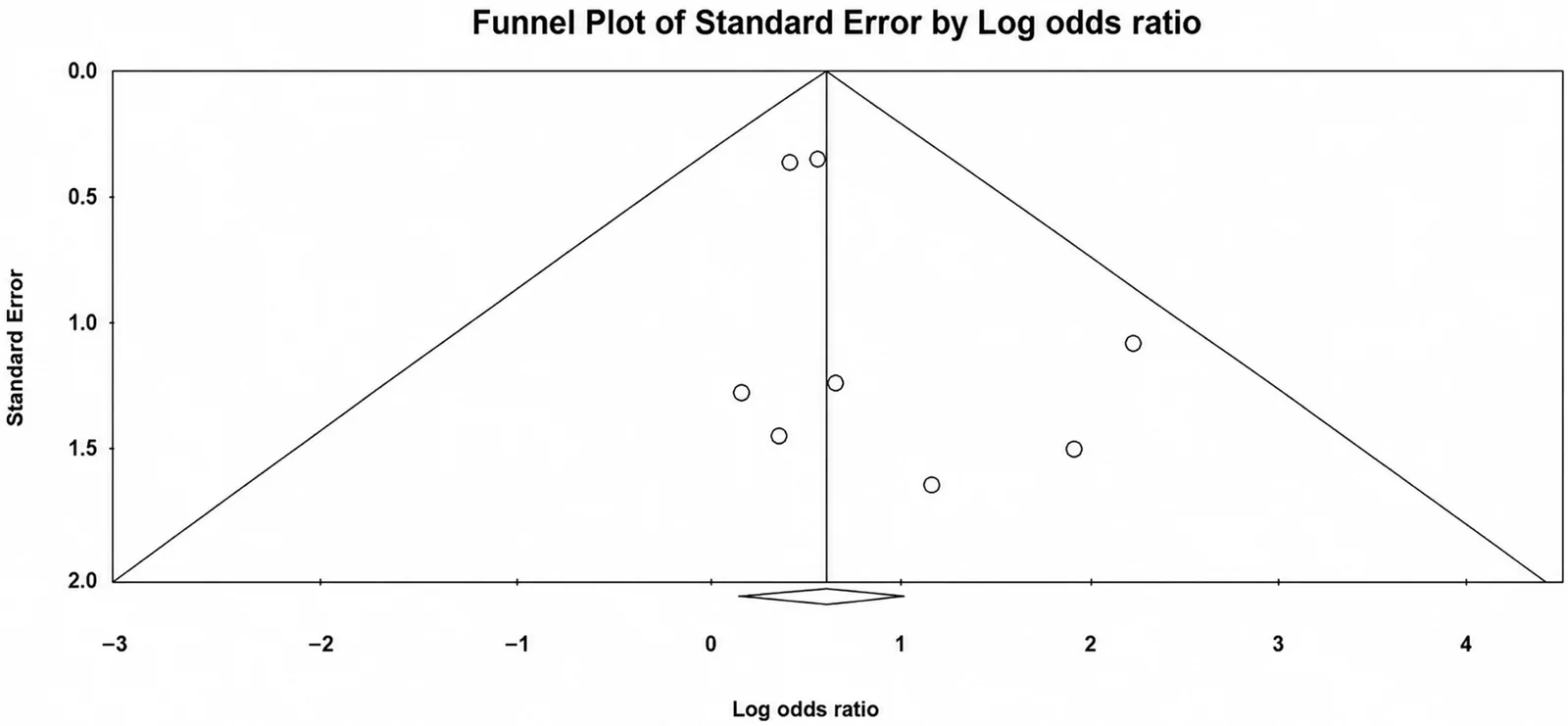
Funnel plot assessing potential publication bias in the meta-analysis of anxiety-related outcomes among patients with mitral valve prolapse.

Potential publication bias and small-study effects were evaluated by visual inspection of the funnel plot (Figure 3) and by Egger’s regression test.

Visual examination of the funnel plot revealed no clear evidence of marked asymmetry, although some imbalance among smaller studies was observed and the limited number of studies precluded a reliable visual assessment of funnel plot symmetry.

Egger’s regression analysis did not detect statistically significant funnel plot asymmetry (intercept 0.590, standard error 0.407, 95% CI −0.407 to 1.586, two-tailed *p* = 0.198). However, because only eight studies were included in the quantitative synthesis, both visual inspection of the funnel plot and Egger’s regression had limited ability to detect small-study effects. Accordingly, the non-significant Egger test should not be interpreted as evidence for the absence of publication bias, which could not be reliably assessed in the present meta-analysis.

To explore potential sources of variability in the pooled effect estimate, an exploratory random-effects multivariable meta-regression model was fitted including publication year, proportion of male participants, and mean age as study-level candidate moderators. Given the limited number of studies and the use of aggregate study-level data, this analysis was considered highly exploratory and potentially subject to unstable estimates and ecological bias. The corresponding results are summarized in Table 5.

None of the three study-level moderators reached statistical significance in this exploratory model (publication year, *p* = 0.338; proportion of male participants, *p* = 0.826; mean age, *p* = 0.732). These findings should not be interpreted as evidence that these characteristics do not modify the association between MVP and anxiety disorders, but rather as hypothesis-generating observations requiring confirmation in larger datasets.

Leave-one-out sensitivity analyses showed that the pooled association was not disproportionately influenced by any single study. After sequential exclusion of each investigation, pooled ORs ranged from 1.65 to 2.01, with all corresponding 95% CIs remaining above unity and all associations remaining statistically significant (*p* = 0.009–0.041). No statistical heterogeneity was detected in any iteration (I^2^ = 0%; τ^2^ = 0.000). Detailed results of the leave-one-out sensitivity analysis are provided in Supplementary Materials File S6 [22,23,24,25,26,28,30,31].

### 3.6. Risk of Bias Assessment

Among the 10 studies evaluating anxiety disorders or anxiety-related outcomes in individuals with MVP, one was rated as good methodological quality, eight as fair, and one as poor (Supplementary Materials File S7 [22,23,24,25,26,27,28,29,30,31]). The poor-quality study relied primarily on administrative diagnostic coding and did not contribute to the quantitative meta-analysis.

Among the seven studies evaluating MVP prevalence in individuals with anxiety disorders, one was rated as good methodological quality and six as fair (Supplementary Materials File S8 [32,33,34,35,36,37,38]). Overall, the most frequent methodological limitations included small sample sizes, limited control of potential confounding, and cross-sectional designs precluding assessment of temporal relationships. Accordingly, the predominance of fair-quality evidence warrants caution when interpreting the observed association, particularly with regard to causality and temporality.

### 3.7. Strength and Certainty of the Available Evidence

The certainty of evidence was assessed separately for the two complementary review questions. For the association between MVP and a higher prevalence of anxiety disorders, the observational evidence was initially rated as low certainty and was further downgraded because of methodological limitations, variability in MVP and psychiatric definitions, differences in recruitment settings and populations, and imprecision related to the small sample sizes of several studies. Although the quantitative synthesis showed a statistically significant association, no convincing upgrading factors were identified. Accordingly, the overall certainty of evidence for this association was judged to be very low.

Evidence regarding a higher prevalence of MVP among individuals with anxiety disorders was also judged to be of very low certainty. Findings varied substantially across studies, ranging from markedly increased to similar or lower MVP prevalence compared with control or reference populations, while diagnostic approaches evolved considerably over time. The predominance of observational and cross-sectional designs, heterogeneous psychiatric populations, variable MVP definitions, and limited precision further reduced confidence in this evidence. Therefore, the available data do not establish a causal, temporal, or bidirectional relationship between MVP and anxiety disorders.

## 4. Discussion

### 4.1. Main Findings and Overview of the Available Evidence

The present systematic review provides the first comprehensive synthesis of the available evidence examining both directions of the relationship between MVP and anxiety disorders, complemented by a quantitative meta-analysis of anxiety-related outcomes among patients with MVP versus controls. By integrating findings from studies published over more than four decades, the review offers an updated overview of this long-debated relationship while taking into account the progressive evolution of both echocardiographic diagnostic criteria and psychiatric classification systems.

The systematic review included 17 observational studies, which were analyzed according to two complementary research questions. The first group comprised 10 studies evaluating anxiety-related outcomes among patients with MVP, whereas the second consisted of 7 studies investigating the prevalence of MVP in individuals with anxiety disorders. Overall, the available evidence was characterized by substantial methodological heterogeneity, encompassing differences in study design, participant selection, and sample size, as well as marked variability in both cardiac and psychiatric assessment. MVP was identified using a wide spectrum of diagnostic approaches, ranging from historical M-mode echocardiography [14,15,16], which is known to overestimate disease prevalence [39,40,41], to 2D echocardiographic assessment with variable diagnostic definitions across studies, including contemporary criteria based on current guideline recommendations [5,6], while one nationwide study relied on administrative diagnostic codes [27]. Similarly, psychiatric assessment varied from structured DSM- or ICD-based diagnostic interviews to validated psychometric questionnaires and symptom-specific rating scales. This methodological diversity should be considered when comparing individual studies and represents an important aspect of the available evidence.

Across the 10 studies evaluating anxiety-related outcomes in patients with MVP [22,23,24,25,26,27,28,29,30,31], several reported a higher prevalence of formally diagnosed anxiety disorders or greater anxiety symptom burden compared with control populations, although the strength of the association varied substantially between investigations. Several studies focused specifically on panic disorder, whereas more recent reports evaluated broader anxiety-related manifestations. Conversely, other studies failed to demonstrate significant differences between MVP and control groups, underscoring the variability associated with study populations and psychiatric assessment methods.

The 7 studies assessing the prevalence of MVP among patients with anxiety disorders [32,33,34,35,36,37,38] yielded similarly heterogeneous findings. While several investigations reported a markedly higher prevalence of MVP in patients with panic disorder than in healthy controls or expected population estimates, others observed only modest differences or failed to identify a statistically significant association. Collectively, these findings suggest that the reported relationship between MVP and anxiety disorders has been influenced by differences in patient populations, psychiatric case definitions, and echocardiographic diagnostic approaches over time.

Despite this variability, quantitative synthesis of the eight studies eligible for meta-analysis [22,23,24,25,26,28,30,31] demonstrated a significantly higher likelihood of anxiety-related outcomes among patients with MVP, with a pooled OR of 1.78. No statistical between-study heterogeneity was detected (I^2^ = 0%); however, this finding should not be interpreted as evidence of clinical or methodological equivalence across studies. The included investigations differed substantially in recruitment setting, participant age, echocardiographic definition of MVP, psychiatric constructs, and methods used to ascertain anxiety-related outcomes, ranging from formal psychiatric diagnoses to symptom-based or psychometric assessments. Moreover, with only eight studies contributing to the quantitative synthesis, the statistical power to detect heterogeneity was limited. Leave-one-out sensitivity analyses showed that the direction of the pooled association was not disproportionately influenced by any single study. However, publication bias could not be reliably assessed because only eight studies contributed to the quantitative synthesis, limiting the interpretability of both funnel plot inspection and Egger’s regression.

Overall, the findings support a statistically significant association between MVP and anxiety-related outcomes, but their clinical interpretation requires consideration of the substantial methodological diversity and very low certainty of the underlying evidence.

### 4.2. Pathophysiological Mechanisms Linking Mitral Valve Prolapse and Anxiety Disorders

The mechanisms underlying the association between MVP and anxiety disorders remain incompletely defined and are probably multifactorial. Rather than supporting a simple causal relationship, the available evidence suggests a potentially reciprocal interplay among autonomic imbalance, cardiopulmonary symptom generation, altered interoceptive processing, hyperventilation, and psychological responses. Such mechanistic reciprocity remains hypothetical and should not be interpreted as evidence of an established bidirectional epidemiological relationship between MVP and anxiety disorders. Accordingly, the higher prevalence of anxiety disorders observed in the present meta-analysis should not be interpreted as evidence that MVP directly causes psychiatric disease in all affected individuals.

Autonomic nervous system dysregulation represents one of the most frequently proposed mechanisms. Symptomatic patients with MVP may exhibit increased sympathetic activity and reduced parasympathetic modulation, resulting in tachycardia, palpitations, orthostatic symptoms, exaggerated cardiovascular reactivity, and heightened adrenergic responses during emotional or physical stress [42,43,44,45]. A similar physiological pattern is frequently observed in panic disorder and other anxiety conditions [46,47]. In symptomatic children with MVP, diminished vagal activity and increased sympathetic tone have also been described, supporting a possible neuroautonomic overlap between the cardiac and psychiatric phenotypes [48,49].

Altered interoceptive sensitivity may further amplify this relationship [50,51]. Patients with MVP can experience palpitations, chest discomfort, dyspnea, dizziness, or forceful cardiac contractions despite only mild leaflet abnormalities, trivial mitral regurgitation, or preserved conventional ventricular function. Increased attention to internal bodily sensations may lead to catastrophic interpretation of otherwise benign symptoms, thereby promoting anticipatory anxiety, fear of cardiac events, and recurrent panic manifestations. This mechanism may help explain why anxiety burden sometimes correlates more closely with symptoms and perceived illness severity than with the objective anatomical severity of MVP.

Hyperventilation may represent an additional link between autonomic activation, respiratory symptoms, and anxiety [52,53]. Anxiety-related overbreathing can produce hypocapnia, dizziness, paresthesia, chest tightness, dyspnea, and palpitations, reproducing many of the complaints traditionally attributed to MVP syndrome [54,55]. Hyperventilation may also alter ventricular repolarization through diaphragmatic displacement of the heart, changes in autonomic tone, catecholamine-mediated asynchronous repolarization, and transient changes in extracellular potassium [56,57]. These effects can generate pseudo-ischemic ST-segment depression, particularly during exercise, and have been specifically reported in patients with MVP [58,59]. A false-positive exercise test may subsequently reinforce concern about occult cardiac disease, initiate further investigations, and intensify health-related anxiety even when coronary disease is ultimately excluded.

Psychological and behavioral mechanisms are therefore likely to sustain a self-reinforcing cycle. Palpitations, atypical chest pain, dyspnea, dizziness, and exercise intolerance may prompt repeated medical consultations and diagnostic testing. Persistent uncertainty regarding their origin can strengthen illness perception and symptom monitoring, particularly when complaints appear disproportionate to the degree of mitral dysfunction. Conversely, pre-existing anxiety may increase symptom reporting, healthcare utilization, and the probability of undergoing echocardiography, thereby contributing to referral and detection bias [60].

An additional hypothesis-generating mechanism concerns gastroesophageal reflux disease (GERD) and increased intra-abdominal pressure. Neither GERD nor intra-abdominal pressure was directly evaluated in the studies included in the present review, and their potential contribution to the MVP–anxiety relationship therefore remains speculative. Increased intra-abdominal pressure, including that occurring during pregnancy, may promote gastroesophageal reflux [61,62], while GERD has independently been associated with anxiety and may generate symptoms such as non-cardiac chest pain and dyspnea that overlap with those commonly reported by symptomatic patients with MVP [63]. Pregnancy additionally induces cardiovascular adaptations that may increase awareness of palpitations and other cardiopulmonary sensations [64]. These observations provide a possible rationale for future investigation but do not establish GERD or increased intra-abdominal pressure as mediators of the association observed in the present meta-analysis.

### 4.3. Thoracic Geometry as a Potential Extrinsic Link Between Mitral Valve Prolapse and Anxiety

Thoracic geometry represents a potential, but currently unproven, extrinsic factor that may contribute to the clinical overlap between MVP and anxiety-related symptoms. Importantly, thoracic morphology was not directly evaluated as a mediator in the studies included in the present systematic review or meta-analysis; the following considerations should therefore be regarded as hypothesis-generating and based on complementary evidence from the literature. Accordingly, this section is intended to identify a potential direction for future research rather than to provide a mechanistic explanation for the meta-analytic association observed in the present study.

MVP has been reported to occur more frequently in individuals with thoracic skeletal abnormalities, including pectus excavatum, scoliosis, and straight back syndrome [65], providing a rationale for exploring thoracic morphology as a potential modifier of the MVP clinical phenotype.

A reduced antero-posterior (A-P) thoracic diameter may alter the spatial relationship between the sternum, heart, and vertebral column and can be quantified using the modified Haller index (MHI), calculated as the ratio of the maximum external latero-lateral thoracic diameter, obtained using a rigid ruler coupled to a level, to the minimum internal A-P thoracic diameter measured by transthoracic echocardiography [66]. Such anatomical constraint has been proposed to influence cardiac position and mechanics and could potentially contribute to palpitations, chest discomfort, or heightened perception of cardiac motion. Supporting this possibility, although in a different clinical setting, studies in patients with persistent atrial fibrillation found an association between higher MHI and symptomatic presentation [67,68]. These observations cannot be directly extrapolated to MVP-associated anxiety but provide a rationale for future investigation.

Thoracic configuration may also interact with exercise-related phenomena. Mechanical cardiac constraint and altered ventricular motion have been associated with pseudo-ischemic electrocardiographic changes; in a patient with pectus excavatum, exercise-induced inferolateral ST-segment depression occurred despite normal coronary anatomy and negative myocardial perfusion imaging and was accompanied by abnormal myocardial mechanics [69]. Whether similar mechanisms contribute to symptom perception or anxiety among patients with MVP remains unknown.

A complementary psychosocial pathway may arise from the impact of visible chest wall abnormalities on body image and social functioning [70,71]. In patients referred for pectus excavatum repair, social anxiety symptoms and impaired psychological well-being have been reported [72], while a larger adult cohort demonstrated frequent anxiety, depression, avoidance of activities exposing the chest, and distress related to chest appearance [73]. These observations concern populations with chest wall deformities rather than patients specifically selected for MVP and therefore provide indirect evidence only.

**Figure 4:**
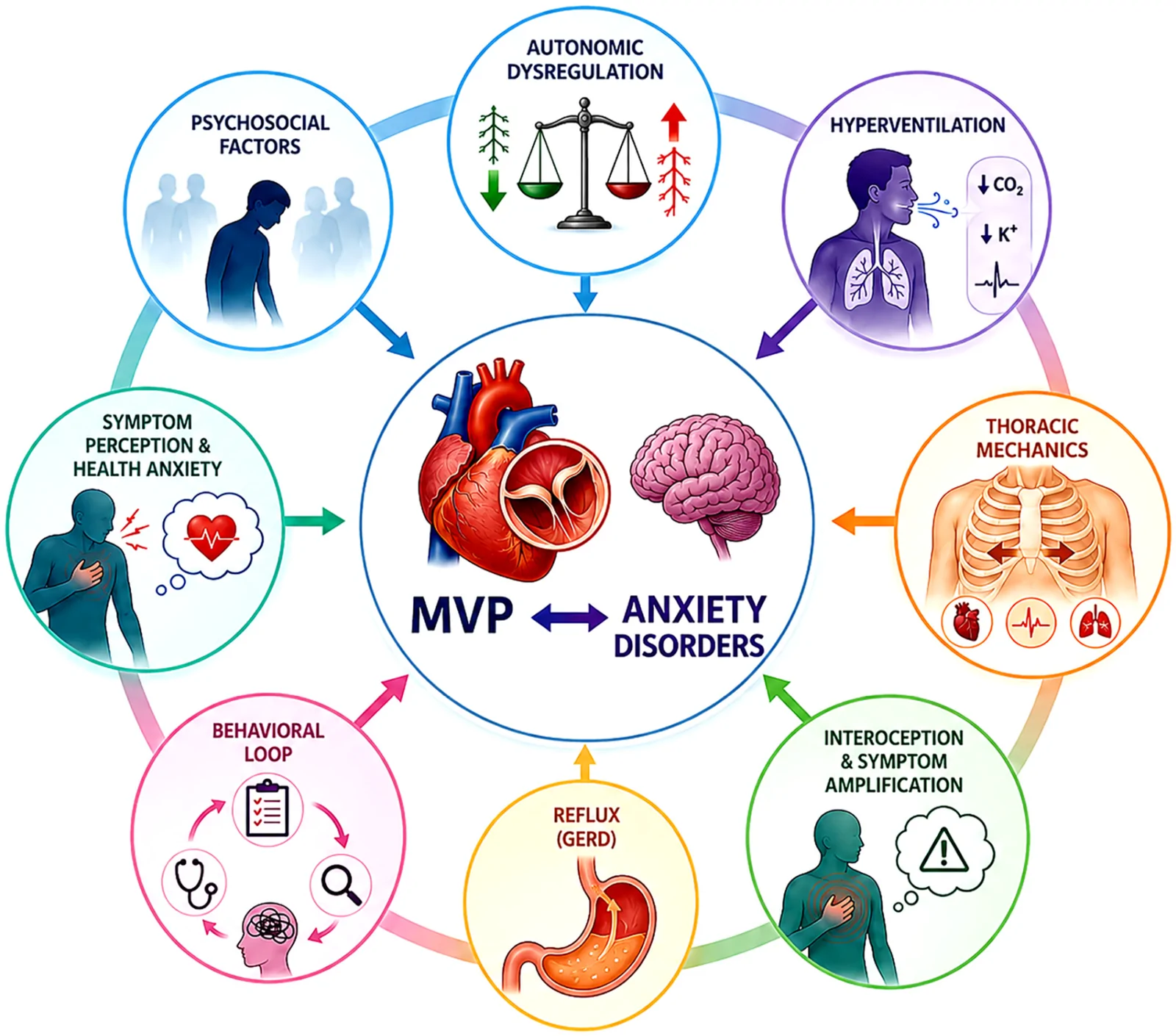
Proposed pathophysiological mechanisms potentially linking mitral valve prolapse (MVP) and anxiety disorders. Schematic representation of a hypothesis-generating multifactorial framework of potential mechanisms that may contribute to the observed association between MVP and anxiety-related outcomes. These mechanisms were not directly evaluated in the present meta-analysis and should not be interpreted as established causal pathways.

Taken together, these findings suggest a testable thoracic-geometry hypothesis involving potential mechanical and psychosocial pathways. However, current evidence does not establish that thoracic geometry mediates the association between MVP and anxiety disorders, and prospective studies incorporating standardized thoracic morphometry and psychiatric assessment are required before clinical or risk-stratification implications can be established. The broader multifactorial framework, including autonomic dysregulation, altered interoception, hyperventilation, thoracic geometry, GERD-related mechanisms, and psychosocial influences, is summarized in Figure 4 and should likewise be interpreted as hypothesis-generating.

### 4.4. Clinical Implications and Future Perspectives

The findings of the present systematic review and meta-analysis have potential clinical implications. The observed association between MVP and anxiety disorders suggests that psychological symptoms deserve consideration in patients with MVP, particularly when palpitations, atypical chest pain, dyspnea, dizziness, or panic-like manifestations appear disproportionate to the severity of valvular disease. Conversely, cardiovascular evaluation may be appropriate in patients with anxiety disorders and prominent cardiac symptoms when clinical findings raise suspicion of underlying structural heart disease. However, the available evidence does not support routine echocardiographic screening of individuals with anxiety disorders.

Thoracic morphology represents a potential complementary area for future investigation rather than an established component of risk assessment. MVP frequently coexists with thoracic skeletal abnormalities, although its reported prevalence varies according to the specific skeletal phenotype and the diagnostic criteria employed [65,74,75,76]. A previous systematic review reported the highest MVP prevalence in Marfan syndrome (47.3%), followed by pectus excavatum (30.8%), scoliosis (26.3%), straight back syndrome (25.5%), and pectus carinatum (23%) [65]. Importantly, these estimates should be interpreted in the context of the evolution of echocardiographic diagnosis: studies conducted before 2000 reported approximately two-fold higher MVP prevalence than more recent investigations, likely reflecting the lower specificity of historical diagnostic criteria [65]. Indeed, the studies cited here were methodologically heterogeneous, with Udoshi et al. using M-mode echocardiography [74], whereas Nouh et al. [75] and Movahed et al. [76] employed 2D echocardiographic assessment. Therefore, differences in reported MVP prevalence across thoracic phenotypes cannot be attributed solely to the underlying skeletal abnormality.

The MHI provides a non-invasive, radiation-free method for quantifying thoracic geometry. The original MHI methodology was validated in a single-center prospective study of 100 subjects against the conventional Haller Index derived from two-plane chest radiography, showing strong correlation and close agreement between the two approaches [66]. However, direct validation against computed tomography (CT) or magnetic resonance imaging (MRI) and independent multicenter validation have not yet been completed. Moreover, although a recently developed patented anatomical measurement device may facilitate a more standardized bedside acquisition of thoracic dimensions, its learning curve, measurement accuracy, and inter- and intra-observer reproducibility have not yet undergone formal prospective validation. These aspects, together with direct comparison against CT- or MRI-derived thoracic measurements, represent important objectives for future studies.

Thoracic morphology has also been associated with alterations in cardiac geometry and myocardial mechanics across different cardiovascular settings. In particular, a narrow A-P thoracic diameter may exert an extrinsic mechanical constraint on the heart, altering cardiac position and systolic motion and potentially inducing a dynamic pattern of myocardial wall-motion dyssynergy that becomes more pronounced during physical exercise. These mechanically induced wall-motion abnormalities, including paradoxical septal motion and regional hypokinetic or dyskinetic patterns, may mimic the regional hypokinesia or akinesia typically associated with inducible myocardial ischemia due to obstructive coronary artery disease, thereby potentially leading to false-positive stress echocardiographic findings [77].

Nevertheless, current evidence is insufficient to establish that any specific thoracic or spinal phenotype identifies patients at increased cardiovascular risk or to recommend routine thoracic morphometry for identifying MVP patients at increased risk of anxiety disorders.

Future prospective studies should determine whether standardized thoracic morphometry provides incremental information when integrated with contemporary MVP criteria, rhythm assessment, autonomic evaluation, myocardial deformation imaging, exercise testing, and standardized psychiatric assessment. Such studies are needed to establish whether thoracic geometry contributes independently to the MVP–anxiety relationship and whether its assessment has meaningful clinical or risk-stratification value.

### 4.5. Strengths, Methodological Considerations, and Study Limitations

The present systematic review has several important strengths. To the best of our knowledge, this is the first study to comprehensively synthesize the evidence addressing both directions of the relationship between MVP and anxiety disorders by integrating investigations evaluating anxiety-related outcomes among patients with MVP and the prevalence of MVP among individuals with anxiety disorders. Furthermore, only studies including an appropriate control population were incorporated into the quantitative synthesis, thereby providing a more reliable estimate of the overall association. The absence of detectable statistical heterogeneity in the primary meta-analysis, together with the leave-one-out sensitivity analyses, indicates that the pooled association was not disproportionately influenced by any single study, although these findings should be interpreted in the context of the clinical and methodological diversity of the included investigations.

Nevertheless, several methodological limitations should be acknowledged when interpreting the available evidence. Importantly, the absence of detectable statistical heterogeneity (I^2^ = 0%) should not be interpreted as evidence of clinical or methodological homogeneity, given the substantial differences across studies in participant age, pediatric versus adult populations, referral setting, comparator groups, psychiatric phenotype and severity, and MVP ascertainment.

Most included investigations were single-center observational studies with relatively small sample sizes, and only one nationwide population-based study was identified. Consequently, residual selection bias and unmeasured confounding cannot be completely excluded. Moreover, the literature spans more than four decades, during which substantial changes occurred in both the diagnosis of MVP and the classification of anxiety disorders.

An important source of methodological heterogeneity concerns the echocardiographic definition of MVP. Earlier investigations primarily relied on M-mode echocardiography, a technique now recognized to overestimate MVP prevalence because of its one-dimensional imaging approach and limited appreciation of the three-dimensional saddle-shaped geometry of the mitral annulus. In contrast, more recent studies predominantly used 2D echocardiographic assessment, although the specific diagnostic definitions varied and did not uniformly correspond to the current criterion of systolic leaflet displacement ≥2 mm beyond the mitral annular plane in the parasternal long-axis view. One nationwide epidemiological study identified MVP through administrative diagnostic codes rather than direct echocardiographic confirmation [27]. These differences may have contributed to variability in patient classification across studies.

Similarly, considerable heterogeneity was observed in the psychiatric assessment of anxiety disorders. The included investigations employed a wide range of diagnostic approaches, including structured DSM- or ICD-based psychiatric interviews, clinician-administered diagnostic instruments, and multiple validated psychometric questionnaires evaluating panic disorder, generalized anxiety, separation anxiety, anxiety sensitivity, or global anxiety symptoms. Consequently, the psychiatric outcomes evaluated across studies were not entirely equivalent and may represent different clinical constructs within the anxiety disorder spectrum.

Additional limitations include differences in study populations, participant age, referral setting, symptom burden, and the severity of associated mitral regurgitation, all of which may have influenced the occurrence and severity of anxiety-related outcomes. Furthermore, because aggregate study-level rather than individual patient-level data were available, adjustment for potentially important confounding variables—including autonomic dysfunction, connective tissue disorders, medication use, and psychiatric treatment—was not possible.

Finally, the limited number of studies eligible for quantitative synthesis precluded more extensive subgroup analyses and rendered the exploratory multivariable meta-regression substantially underpowered and potentially unstable; moreover, the use of aggregate study-level moderators introduces the possibility of ecological bias.

Despite these limitations, the association observed across the complementary analyses warrants further investigation in large, prospective, multicenter studies employing standardized echocardiographic diagnostic criteria and uniform psychiatric assessment methods.

## 5. Conclusions

This systematic review and meta-analysis demonstrates a significant association between MVP and an increased prevalence of anxiety-related outcomes compared with individuals without MVP. However, the pooled estimate was derived from a small number of clinically and methodologically diverse observational studies, predominantly of fair methodological quality, and should not be interpreted as evidence of a temporal or causal relationship. Evidence addressing the prevalence of MVP among individuals with anxiety disorders remains heterogeneous and currently supports descriptive rather than quantitative synthesis.

Residual confounding by clinical, cardiovascular, and referral-related factors cannot be excluded. The mechanisms underlying the observed association remain uncertain, with thoracic geometry representing a plausible but still hypothesis-generating contributor to the interplay between MVP, symptom burden, and psychological distress. Larger prospective studies using contemporary MVP criteria, standardized psychiatric assessment, and comprehensive phenotyping, including quantitative thoracic morphometry, are needed to clarify the direction, mechanisms, and clinical significance of this association.

## Figures and Tables

**Table 1:** Methodological and clinical characteristics of studies investigating anxiety disorders among patients with mitral valve prolapse [22,23,24,25,26,27,28,29,30,31].

Study Name (Publication Year), Country	Study Design	Study Population	Psychiatric Assessment	MVP Diagnostic Criteria	Main Findings
Mazza D.L. (1986), USA [22]	Prospective, single-center case–control	48 MVP vs. 49 controls	DSM-III, ZAS, ZDS	M-mode	No ↑ anxiety/panic in MVP
Bowen R.C. (1991), Canada [23]	Prospective, single-center case–control	19 MVP vs. 26 controls	DIS, SCL-90-R	M-mode	No ↑ panic/anxiety in MVP
Tamam L. (2000), Turkey [24]	Prospective, single-center case–control	50 MVP, 50 PD, 50 controls	SCID DSM-IV, SCL-90-R, BDI, STAI	M-mode	↑ Panic disorder in idiopathic MVP
Lung F.W. (2008), Taiwan [25]	Prospective, single-center observational	Young male outpatients with chest pain	MINI	2D echo	MVP is not a risk factor for panic disorder
Orhan A.L. (2009), Turkey [26]	Prospective, single-center cross-sectional case–control	33 MVP vs. 14 controls	SSAS, SCAS	2D echocardiography (ASE criteria)	MVP syndrome showed ↑ anxiety
Dubey N.K. (2016) Taiwan [27]	Nationwide retrospective database study	NHIRD population	ICD-9-CM	ICD-9-CM	Association strongest in young women
Bayer-Topilsky T. (2016) Israel [28]	Prospective, single-center cohort	216 MVP vs. 65 non-MVP	HADS, PCL-C, SF-12, LASA	Comprehensive 2D echocardiography	No independent association
Çağlayan U. (2021) Turkey [29]	Prospective, single-center cross-sectional	2550 school children	Clinical symptom questionnaire	2D echo	Anxiety was the most common reported symptom (37.5%); no control comparison
Beyazal M. (2023) Turkey [30]	Prospective, single-center case–control	115 MVP vs. 53 controls	SCARED	2D echo	No higher overall anxiety; symptomatic MVP showed higher anxiety scores
Yetkin Z.D. (2026) Turkey [31]	Prospective, single-center case–control	65 MVP vs. 60 controls	K-SADS-PL, SAD Scale, CASI	2D echocardiography (ASE criteria)	↑ Separation anxiety in youth with MVP

The table summarizes the study design, study population, psychiatric assessment methods, echocardiographic criteria used for MVP diagnosis, and the principal findings of each included investigation. ASE, American Society of Echocardiography; BDI, Beck Depression Inventory; CASI, Childhood Anxiety Sensitivity Index; DIS, Diagnostic Interview Schedule; DSM, Diagnostic and Statistical Manual of Mental Disorders; HADS, Hospital Anxiety and Depression Scale; ICD-9-CM, International Classification of Diseases, Ninth Revision, Clinical Modification; K-SADS-PL, Schedule for Affective Disorders and Schizophrenia for School-Age Children–Present and Lifetime Version; LASA, Linear Analogue Self-Assessment; MINI, Mini International Neuropsychiatric Interview; MVP, mitral valve prolapse; NHIRD, National Health Insurance Research Database; PCL-C, PTSD Checklist–Civilian Version; SCAS, State–Trait Cardiac Anxiety Scale; SCID, Structured Clinical Interview for DSM Disorders; SCL-90-R, Symptom Checklist-90-Revised; SSAS, Somatosensory Amplification Scale; STAI, State–Trait Anxiety Inventory; ZAS, Zung Self-Rating Anxiety Scale; ZDS, Zung Self-Rating Depression Scale; ↑, increased.

**Table 2:** Methodological and clinical characteristics of studies investigating mitral valve prolapse among patients with anxiety disorders [32,33,34,35,36,37,38].

Study Name (Publication Year), Country	Study Design	Study Population	Psychiatric Assessment	MVP Diagnostic Criteria	Main Findings
Crowe R.R. (1979), USA [32]	Prospective, single-center case–control	20 anxiety neurosis vs. 20 controls	Feighner criteria	M-mode echocardiography	Reduced exercise tolerance only in anxiety patients with MVP
Venkatesh A. (1980), USA [33]	Prospective, single-center cross-sectional	21 panic disorder patients	Feighner criteria	M-mode echocardiography	High prevalence of MVP in panic disorder
Shear M.K. (1984) USA [34]	Prospective, single-center cross-sectional	25 panic disorder patients	DSM-III	M-mode echocardiography (stringent criteria)	MVP prevalence (8%) comparable to the general population
Liberthson R. (1986) USA [35]	Prospective, single-center cross-sectional	131 panic disorder/ agoraphobia	DSM-III	Comprehensive 2D echocardiography	34% definite MVP (39% definite/probable MVP)
Carney R.M. (1990) USA [36]	Prospective, single-center cross-sectional	100 chest pain patients without CAD	Structured psychiatric interview (DSM-III)	Comprehensive 2D echocardiography	MVP associated with panic disorder and major depression in patients with non-CAD chest pain
Toren P. (1999) Israel [37]	Prospective, single-center case–control	52 children with anxiety vs. 51 controls	K-SADS, RCMAS	Blinded M-mode + 2D echocardiography	No increased prevalence of MVP in childhood anxiety disorders
Filho A.S. (2011), Brazil [38]	Prospective, single-center case–control	41 panic disorder, 89 SAD, 102 controls	SCID	2D echocardiography (current standardized criteria)	No association between panic disorder or social anxiety disorder and MVP

The table summarizes the study design, study population, psychiatric diagnostic methods, echocardiographic criteria applied for MVP identification, and the principal findings of each investigation. CAD, coronary artery disease; DSM-III, Diagnostic and Statistical Manual of Mental Disorders, Third Edition; K-SADS, Schedule for Affective Disorders and Schizophrenia for School-Age Children; MVP, mitral valve prolapse; RCMAS, Revised Children’s Manifest Anxiety Scale; SAD, social anxiety disorder; SCID, Structured Clinical Interview for DSM Disorders; 2D, two-dimensional.

**Table 3:** Weighted summary of demographic, clinical, and psychiatric characteristics in patients with mitral valve prolapse and control subjects.

Variable	MVP (Participants Included)	Controls (Participants Included)
Av. age, years	28.4 (590)	29.5 (2862)
Av. BMI, kg/m^2^	21.7 (281)	23.5 (2597)
Av. SBP, mmHg	109 (248)	116.1 (2583)
Av. HR, bpm	91.7 (65)	93.3 (2532)
Av. LVEF, %	64.1 (216)	64.0 (65)
Av. sPAP, mmHg	32.9 (216)	33.6 (65)
Av. SCARED score	29.2 (115)	24.9 (53)
Av. DSM-5 SAD Severity Scale	13 (65)	5 (60)
Av. EMBU-P overprotection score	23.6 (65)	21.1 (60)
Av. SCL-90-R anxiety score	60.3 (19)	54.8 (26)
Av. SSAS score	32.6 (33)	14.9 (14)
Av. SCAS score	31.3 (33)	16.9 (14)
Av. HADS-A score	5.1 (216)	4.3 (65)
Av. Zung Anxiety Scale score	35.9 (48)	30.8 (49)
Anxiety/panic disorder prevalence, %	16.1 (13,271)	11.3 (344)

Continuous variables are presented as sample size-weighted mean values, whereas categorical variables are reported as weighted percentages based on the available aggregate study-level data. The number of participants contributing to each variable is reported in parentheses. Because the contributing studies and sample sizes differed substantially across variables and between groups, these values are presented exclusively as descriptive summaries and should not be interpreted as patient-level estimates or formal between-group comparisons. No inferential statistical testing was performed for the descriptive data presented in this table.

**Table 4:** Weighted summary of demographic and clinical characteristics of patients with anxiety disorders evaluated for mitral valve prolapse.

Variable	Weighted Value
Total patients with anxiety/panic disorders, *n*	672
Male sex, %	39.2
Mean age, years	28.2
Patients with MVP, *n* (%)	89 (13.2)
Male sex among MVP patients, %	33.3
Mean age of MVP patients, years	36.5

Continuous variables are presented as sample size-weighted mean values and categorical variables as aggregate descriptive proportions based on available study-level data. The crude aggregate prevalence of MVP was calculated by dividing the total number of patients with MVP by the total number of anxiety/panic disorder participants across contributing studies. These estimates are descriptive and should not be interpreted as formal meta-analytic pooled estimates.

**Table 5:** Random-effects meta-regression evaluating the potential influence of selected study-level demographic and methodological variables on the pooled association between mitral valve prolapse and anxiety-related outcomes. Regression coefficients represent the estimated change in the logarithm of the odds ratio associated with each moderator. For each covariate, the regression coefficient, standard error, 95% confidence interval (95% CI), and two-sided *p* value are reported.

Covariate	Coefficient	Standard Error	95% Lower	95% Upper	2-Sided *p*-Value
Intercept	58.464	60.060	−59.250	176.179	0.330
Publication year	−0.029	0.030	−0.087	0.030	0.338
Male sex	−0.004	0.017	−0.038	0.030	0.826
Mean age	−0.006	0.016	−0.037	0.026	0.732

## Data Availability

The dataset generated from the extraction of data from the included studies will be made openly accessible through Zenodo (https://zenodo.org; accessed 7 July 2026).

## Supplementary Materials

The following supporting information can be downloaded at: https://www.mdpi.com/article/10.3390/jcm15176792/s1, File S1: PRISMA 2020 checklist; File S2: The full INPLASY record (https://inplasy.com/inplasy-2026-8-0006/, accessed on 1 August 2026); File S3: Complete database-specific search strategies for PubMed, Scopus, and EMBASE, including Boolean operators, search fields, limits, and date of the final search; File S4: Bibliographic details of potentially relevant reports that could not be retrieved for full-text eligibility assessment; File S5: Study-level psychiatric endpoints, assessment methods, and event and denominator data used for the quantitative meta-analysis [22,23,24,25,26,28,30,31]; File S6: Leave-one-out sensitivity analysis reporting pooled ORs, 95% CIs, *p* values, I^2^, and τ^2^ after sequential exclusion of each study [22,23,24,25,26,28,30,31]; File S7: Design-specific methodological quality and risk-of-bias assessment of studies evaluating anxiety disorders or anxiety-related outcomes in individuals with MVP [22,23,24,25,26,27,28,29,30,31]; File S8: Design-specific methodological quality and risk-of-bias assessment of studies evaluating MVP prevalence among individuals with anxiety disorders [32,33,34,35,36,37,38].
