# Supplementary material for: Mitral Valve Prolapse and Anxiety Disorders: A Systematic Review of Evidence in Both Directions and Meta-Analysis
Source: J Clin Med. 2026 Sep 1;15(17):6792. doi: 10.3390/jcm15176792 (PMC13566358; doi:10.3390/jcm15176792)
Supplement: Supplementary file 1 [file jcm-15-06792-s001.zip › Supplementary Material S2.pdf]

International Platform of Registered Systematic Review and Meta-analysis Protocols

INPLASY

Bidirectional Associations Between Mitral Valve Prolapse and Anxiety Disorders: A Systematic Review and Meta-Analysis

INPLASY202680006
doi: 10.37766/inplasy2026.8.0006
Received: 1 August 2026
Published: 1 August 2026

Sonaglioni, A; Gramaglia, GF; Nicolosi, GL; Baravelli, M; Lombardo, M.

Corresponding author:
Andrea Sonaglioni

sonaglioniandrea@gmail.com

Author Affiliation:
MultiMedica IRCCS.

ADMINISTRATIVE INFORMATION

Support - No funding.

Review Stage at time of this submission - Data analysis.

Conflicts of interest - None declared.

INPLASY registration number: INPLASY202680006

Amendments - This protocol was registered with the International Platform of Registered Systematic Review and Meta-Analysis Protocols (INPLASY) on 1 August 2026 and was last updated on 1 August 2026.

INTRODUCTION

Review question / Objective To systematically evaluate the available evidence on the relationship between mitral valve prolapse (MVP) and anxiety disorders by examining both directions of the association: the occurrence of anxiety disorders in individuals with MVP and the prevalence of MVP among patients with anxiety disorders. In addition, this review aims to quantify the risk of anxiety disorders in patients with MVP through meta-analysis of comparative studies and to investigate whether differences in echocardiographic diagnostic criteria influence the observed association.

Rationale The potential relationship between mitral valve prolapse (MVP) and anxiety disorders has been debated for decades, with published studies yielding inconsistent and sometimes contradictory findings. Differences in study design, patient populations, psychiatric diagnostic methods, and evolving echocardiographic definitions of MVP have contributed to uncertainty regarding the existence and magnitude of this association. While some investigations have suggested that anxiety disorders occur more frequently in patients with MVP, others have failed to confirm this observation using contemporary diagnostic criteria. Likewise, several studies have explored the prevalence of MVP among individuals with anxiety disorders, but the available evidence has never been comprehensively synthesized. A systematic review with quantitative analysis is therefore needed to critically appraise the current literature, estimate the strength of the association where appropriate, and explore whether changes in MVP diagnostic criteria may explain the heterogeneity of published results.

Condition being studied This review focuses on the association between mitral valve prolapse (MVP), the most common primary valvular abnormality, and anxiety disorders, including panic disorder and related anxiety-spectrum conditions. Specifically, it examines whether individuals with MVP have an increased prevalence of anxiety disorders compared with control populations and,

Sonaglioni et al. INPLASY protocol 202680006. doi:10.37766/inplasy2026.8.0006 Downloaded from https://inplasy.com/inplasy-2026-8-0006/

conversely, whether patients with anxiety disorders are more likely to have MVP. Particular attention is given to the potential impact of different echocardiographic diagnostic criteria, including historical M-mode and contemporary two-dimensional definitions, on the reported associations. The review also explores the clinical and methodological factors that may account for inconsistencies across the published literature.

METHODS

Search strategy A comprehensive electronic search will be conducted in PubMed, Scopus, and EMBASE from database inception to July 2026, without restrictions on language, publication date, or geographic location. The search strategy will combine controlled vocabulary (e.g., MeSH terms) and free-text keywords related to mitral valve prolapse and anxiety disorders using Boolean operators ("AND" and "OR"). Search terms will include, but will not be limited to: "mitral valve prolapse", "mitral valve disorder", "anxiety", "anxiety disorders", "panic disorder", "panic attack", "phobia", "agoraphobia", "social anxiety disorder", "separation anxiety", "depression", and "echocardiography". The reference lists of all eligible articles and relevant review papers will also be manually screened to identify additional studies that may have been missed during the electronic search. Two reviewers will independently perform the literature search, study selection, and eligibility assessment, with disagreements resolved through discussion and, when necessary, consultation with a third reviewer.

Participant or population Individuals of any age or sex included in observational studies evaluating the relationship between mitral valve prolapse (MVP) and anxiety disorders. Eligible populations comprise patients with echocardiographically confirmed MVP, individuals diagnosed with anxiety disorders or panic disorder according to validated psychiatric criteria, and appropriate control groups without the condition of interest. Both adult and pediatric populations, irrespective of clinical setting or geographic region, will be considered for inclusion.

Intervention No therapeutic intervention will be evaluated. The exposure of interest is the presence of mitral valve prolapse (MVP), diagnosed by echocardiography using either historical M-mode or contemporary two-dimensional criteria, and/or the presence of anxiety disorders diagnosed according to validated psychiatric assessment tools or internationally recognized diagnostic classifications (e.g., DSM- or ICD-based criteria). The review will examine the bidirectional association between these two conditions.

Comparator Comparator groups will consist of individuals without mitral valve prolapse when evaluating the prevalence of anxiety disorders in patients with MVP, and individuals without anxiety disorders when assessing the prevalence of MVP among patients with anxiety disorders. Studies including healthy controls, population-based controls, or other appropriate non-exposed comparison groups will be eligible. The comparator definitions adopted in the original studies will be accepted, provided they are clearly described.

Study designs to be included Original observational studies will be considered eligible, including prospective and retrospective cohort studies, case–control studies, cross-sectional studies, and population-based observational investigations. Studies with or without a comparator group will be included in the qualitative systematic review; however, only studies providing appropriate comparative data will be eligible for quantitative meta-analysis. Case reports, case series, conference abstracts, editorials, narrative reviews, systematic reviews, meta-analyses, letters, expert opinions, and clinical practice guidelines will.

Eligibility criteria Studies will be eligible if they are original observational investigations evaluating the association between mitral valve prolapse (MVP) and anxiety disorders in either direction. Specifically, studies must include participants with echocardiographically diagnosed MVP assessed for the presence of anxiety disorders, or individuals with anxiety disorders evaluated for the presence of MVP. MVP diagnosis must be established using clearly defined echocardiographic criteria, including historical M-mode or contemporary two-dimensional approaches. Anxiety disorders should be identified using validated psychometric instruments, structured psychiatric interviews, or internationally recognized diagnostic classifications (e.g., DSM or ICD). No restrictions will be applied regarding participants' age, sex, clinical setting, or country of origin. Studies lacking original data, including reviews, meta-analyses, editorials, conference abstracts, case reports, letters, and clinical practice guidelines, will be excluded. For quantitative synthesis, only studies providing sufficient numerical data to calculate effect estimates or construct contingency tables will be considered. Studies without an appropriate comparison group will contribute only to the qualitative systematic review.

Sonaglioni et al. INPLASY protocol 202680006. doi:10.37766/inplasy2026.8.0006 Downloaded from https://inplasy.com/inplasy-2026-8-0006/

Information sources The literature search will be performed using the electronic databases PubMed, Scopus, and EMBASE, covering all records from database inception through July 2026. In addition to electronic database searches, the reference lists of all included articles and relevant review papers will be manually screened to identify further eligible studies. Where necessary, corresponding authors may be contacted to clarify missing or incomplete data required for study eligibility or quantitative synthesis. No restrictions regarding language, publication year, or country of origin will be applied.

Main outcome(s) The primary outcome will be the association between mitral valve prolapse (MVP) and anxiety disorders, expressed as the odds of anxiety disorders among individuals with MVP compared with control populations. When appropriate, pooled odds ratios (ORs) with corresponding 95% confidence intervals (CIs) will be calculated from comparative studies reporting the number of participants with anxiety disorders in both MVP and control groups. Anxiety disorders identified through validated psychiatric interviews, standardized questionnaires, or internationally accepted diagnostic criteria will be considered eligible outcomes.

Additional outcome(s) Secondary outcomes will include the prevalence of mitral valve prolapse (MVP) among individuals with anxiety disorders, descriptive summaries of demographic and clinical characteristics of the included populations, and comparisons according to the echocardiographic criteria used to diagnose MVP (historical M-mode versus contemporary two-dimensional echocardiography). Additional analyses will explore the influence of study-level characteristics, including publication year, participants' age, and sex distribution, through meta-regression when sufficient data are available. Between-study heterogeneity, publication bias, cumulative evidence over time, and methodological quality of the included studies will also be assessed.

Data management Study selection and data extraction will be performed independently by two reviewers using a standardized data collection form developed specifically for this review. Extracted information will include study characteristics, participant demographics, diagnostic methods for mitral valve prolapse and anxiety disorders, outcome data, and variables required for quantitative synthesis. All extracted data will be cross-checked for accuracy, and any discrepancies will be resolved through discussion or, if necessary, by consultation with a third reviewer. When required, corresponding authors will be contacted to clarify missing or incomplete information. The final dataset will be used for qualitative synthesis and, where appropriate, for meta-analysis using dedicated statistical software.

Quality assessment / Risk of bias analysis The methodological quality of the included studies will be independently evaluated by two reviewers using the National Institutes of Health (NIH) Quality Assessment Tool for Observational Cohort and Cross-Sectional Studies. The tool assesses multiple methodological domains, including study design, participant selection, exposure and outcome ascertainment, control of confounding, and statistical methodology. Each study will be assigned an overall quality rating (Good, Fair, or Poor) according to the NIH guidance. Any disagreements between reviewers will be resolved by discussion or, when necessary, by consultation with a third reviewer. The methodological quality of the included studies will be considered when interpreting the findings of both the qualitative synthesis and the meta-analysis.

Strategy of data synthesis A qualitative synthesis will be undertaken to summarize the characteristics and principal findings of all eligible studies. When sufficient comparative data are available, a quantitative meta-analysis will be performed by calculating pooled odds ratios (ORs) with 95% confidence intervals (CIs). Random-effects models will be used as the primary analytical approach to account for anticipated clinical and methodological variability among studies. Statistical heterogeneity will be evaluated using Cochran's Q test, the I^2 statistic, and τ^2 estimates. Prespecified subgroup analyses will compare studies according to the echocardiographic criteria used for diagnosing mitral valve prolapse (historical M-mode versus contemporary two-dimensional echocardiography). Random-effects meta-regression analyses will be conducted, when appropriate, to investigate the potential influence of study-level characteristics, including publication year, mean age, and sex distribution. Publication bias will be explored through funnel plot inspection and Egger's regression test, and the robustness of the pooled estimates will be examined by sensitivity and cumulative meta-analyses. Statistical analyses will be performed using Comprehensive Meta-Analysis (CMA) software, version 3.0.

Subgroup analysis Where sufficient data are available, predefined subgroup analyses will be performed according to the echocardiographic

Sonaglioni et al. INPLASY protocol 202680006. doi:10.37766/inplasy2026.8.0006 Downloaded from https://inplasy.com/inplasy-2026-8-0006/

method used to diagnose mitral valve prolapse, comparing studies based on historical M-mode criteria with those using contemporary two-dimensional echocardiographic definitions. Additional subgroup analyses may be undertaken according to participant age (adult versus pediatric populations), type of anxiety disorder assessed (e.g., panic disorder versus broader anxiety disorders), and study design, provided that an adequate number of studies is available within each subgroup. These analyses will be conducted to explore potential sources of clinical and methodological heterogeneity and to evaluate the consistency of the pooled findings across different study characteristics.

Sensitivity analysis Sensitivity analyses will be undertaken to assess the robustness and stability of the pooled estimates. A leave-one-out approach will be applied by sequentially excluding each study and recalculating the overall effect size to determine whether any single investigation has a disproportionate influence on the results. Additional sensitivity analyses may be performed by excluding studies considered to be at higher risk of bias or those using historical echocardiographic diagnostic criteria for mitral valve prolapse, when appropriate. The consistency of the findings obtained with random-effects and fixed-effect models will also be examined where applicable.

Language restriction No language restrictions will be applied. Studies published in any language will be considered eligible for inclusion, provided that sufficient information can be obtained for data extraction and methodological assessment.

Country(ies) involved Italy.

Other relevant information This review will be conducted in accordance with the Preferred Reporting Items for Systematic Reviews and Meta-Analyses (PRISMA) 2020 Statement. The review addresses the bidirectional relationship between mitral valve prolapse and anxiety disorders by integrating both qualitative and quantitative evidence from observational studies. Meta-regression, subgroup analyses, cumulative meta-analysis, and publication bias assessment are prespecified to explore potential sources of heterogeneity and the stability of the findings. The review protocol has been developed before completion of the evidence synthesis to enhance methodological transparency and minimize the risk of selective reporting.

Keywords Mitral valve prolapse; Anxiety disorders; Panic disorder; Echocardiography; Systematic review; Meta-analysis; Psychiatric disorders; Two-dimensional echocardiography; M-mode echocardiography; Observational studies.

Dissemination plans The findings of this systematic review and meta-analysis will be disseminated through publication in a peer-reviewed international scientific journal and presentation at national and international cardiology and cardiovascular imaging conferences. The results are expected to contribute to the current evidence regarding the association between mitral valve prolapse and anxiety disorders and to support future clinical research in this field.

Contributions of each author

Author 1 - Andrea Sonaglioni - Author 1 drafted the manuscript.
Email: sonaglioniandrea@gmail.com
Author 2 - Giulio Francesco Gramaglia - The author contributed to the Search Strategy.
Email: giulio.gramaglia@unimi.it
Author 3 - Gian Luigi Nicolosi - The author contributed to the development of the selection criteria, and the risk of bias assessment strategy.
Email: gianluigi.nicolosi@gmail.com
Author 4 - Massimo Baravelli - The author read, provided feedback and approved the final manuscript.
Email: massimo.baravelli@multimedica.it
Author 5 - Michele Lombardo - Supervision.
Email: michele.lombardo@multimedica.it

Sonaglioni et al. INPLASY protocol 202680006. doi:10.37766/inplasy2026.8.0006 Downloaded from https://inplasy.com/inplasy-2026-8-0006/
