# Supplementary material for: Mitral Valve Prolapse and Anxiety Disorders: A Systematic Review of Evidence in Both Directions and Meta-Analysis
Source: J Clin Med. 2026 Sep 1;15(17):6792. doi: 10.3390/jcm15176792 (PMC13566358; doi:10.3390/jcm15176792)
Supplement: Supplementary file 1 [file jcm-15-06792-s001.zip › Supplementary Material S3.pdf]

Database	Search	Search strategy	Search field	Limits	Last search
PubMed	#1	"mitral valve prolapse" AND "anxiety disorders"	Default database search field	None	9 July 2026
PubMed	#2	"mitral valve prolapse" AND "anxiety" AND "prevalence"	Default database search field	None	9 July 2026
PubMed	#3	"mitral valve prolapse" AND "panic disorder"	Default database search field	None	9 July 2026
PubMed	#4	"mitral valve prolapse" AND "agoraphobia"	Default database search field	None	9 July 2026
PubMed	#5	"mitral valve prolapse" AND "depression"	Default database search field	None	9 July 2026
Scopus	#1	"mitral valve prolapse" AND "anxiety disorders"	Default database search field	None	9 July 2026
Scopus	#2	"mitral valve prolapse" AND "anxiety" AND "prevalence"	Default database search field	None	9 July 2026
Scopus	#3	"mitral valve prolapse" AND "panic disorder"	Default database search field	None	9 July 2026
Scopus	#4	"mitral valve prolapse" AND "agoraphobia"	Default database search field	None	9 July 2026
Scopus	#5	"mitral valve prolapse" AND "depression"	Default database search field	None	9 July 2026
EMBASE	#1	"mitral valve prolapse" AND "anxiety disorders"	Default database search field	None	9 July 2026
EMBASE	#2	"mitral valve prolapse" AND "anxiety" AND "prevalence"	Default database search field	None	9 July 2026
EMBASE	#3	"mitral valve prolapse" AND "panic disorder"	Default database search field	None	9 July 2026
EMBASE	#4	"mitral valve prolapse" AND "agoraphobia"	Default database search field	None	9 July 2026
EMBASE	#5	"mitral valve prolapse" AND "depression"	Default database search field	None	9 July 2026
