# Supplementary material for: Mitral Valve Prolapse and Anxiety Disorders: A Systematic Review of Evidence in Both Directions and Meta-Analysis
Source: J Clin Med. 2026 Sep 1;15(17):6792. doi: 10.3390/jcm15176792 (PMC13566358; doi:10.3390/jcm15176792)
Supplement: Supplementary file 1 [file jcm-15-06792-s001.zip › Supplementary Material S4.pdf]

Sivaramakrishnan K, Alexander PJ, Saharsarnamam N. Prevalence of panic disorder in mitral valve prolapse: a comparative study with a cardiac control group. Acta Psychiatr Scand. 1994 Jan;89(1):59-61. doi: 10.1111/j.1600-0447.1994.tb01486.x. PMID: 8140908.

Arfken CL, Lachman AS, McLaren MJ, Schulman P, Leach CN Jr, Farrish GC. Mitral valve prolapse: associations with symptoms and anxiety. Pediatrics. 1990 Mar;85(3):311-5. PMID: 2304784.

Uretsky BF. Does mitral valve prolapse cause nonspecific symptoms? Int J Cardiol. 1982;1(5-6):435-42. doi: 10.1016/0167-5273(82)90069-9. PMID: 7118310.

Chan MP, Hibbert GA, Watkins J. Mitral valve prolapse and anxiety disorders. Br J Psychiatry. 1984 Aug;145:216-7. doi: 10.1192/bjp.145.2.216. PMID: 6466922.

Hall RC, Beresford TP, Popkin MK, Hoffman RS, Wooley B, Tice L, Hall AK. Mitral valve prolapse and anxiety disorders in patients with anorexia nervosa. Psychiatr Med. 1989;7(4):217-33. PMID: 2602568.

Mavissakalian M, Salerni R, Thompson ME, Michelson L. Mitral valve prolapse and agoraphobia. Am J Psychiatry. 1983 Dec;140(12):1612-4. doi: 10.1176/ajp.140.12.1612. PMID: 6650694.

Hamada T, Fukui J, Koshino Y, Murata T, Oomori M, Misawa T, Kuroda M. [Mitral valve prolapse syndrome as an etiologic factor of anxiety disorder]. Rinsho Byori. 1990 Aug;38(8):952-6. Japanese. PMID: 2232260.

Forrest RD. Mitral valve prolapse syndrome in anxiety neurosis. Am Heart J. 1981 Jul;102(1):139. doi: 10.1016/0002-8703(81)90433-6. PMID: 7248047.

Chaleby K, Ziady G. Mitral valve prolapse and social phobia. Br J Psychiatry. 1988 Feb;152:280-1. doi: 10.1192/bjp.152.2.280. PMID: 3167351.

Koshino Y, Izaki K. [Panic disorders and cardiac functions--mitral valve prolapse and electrocardiographic R-R intervals]. Seishin Shinkeigaku Zasshi. 1993;95(11):861-7. Japanese. PMID: 8108527.
