# Supplementary material for: Mitral Valve Prolapse and Anxiety Disorders: A Systematic Review of Evidence in Both Directions and Meta-Analysis
Source: J Clin Med. 2026 Sep 1;15(17):6792. doi: 10.3390/jcm15176792 (PMC13566358; doi:10.3390/jcm15176792)
Supplement: Supplementary file 1 [file jcm-15-06792-s001.zip › Supplementary Material S5.pdf]

Study	Psychiatric endpoint entered in meta-analysis	Diagnostic instrument/framework	MVP events / total	Control events / total
Mazza et al. (1986)	Anxiety/panic disorder	DSM-III psychiatric interview; Zung Anxiety Scale	1/48	0/49
Bowen et al. (1991)	Anxiety/panic disorder	Diagnostic Interview Schedule (DIS)	1/19	1/26
Tamam et al. (2000)	Panic disorder	SCID for DSM-IV Axis I Disorders	8/50	1/50
Lung et al. (2008)	Panic disorder	MINI	1/12	2/27
Orhan et al. (2009)	Study-reported anxiety outcome	SSAS; STAI-State; SCAS/STAI-Trait	6/33	0/14
Bayer-Topilsky et al. (2016)	Study-reported psychoemotional/anxiety outcome	HADS	54/216	12/65
Beyazal et al. (2023)	Study-reported anxiety outcome	SCARED	63/115	22/53
Yetkin et al. (2026)	Separation anxiety disorder	K-SADS-PL-DSM-5; DSM-5 SAD Severity Scale	2/65	1/60
