# Supplementary material for: Mitral Valve Prolapse and Anxiety Disorders: A Systematic Review of Evidence in Both Directions and Meta-Analysis
Source: J Clin Med. 2026 Sep 1;15(17):6792. doi: 10.3390/jcm15176792 (PMC13566358; doi:10.3390/jcm15176792)
Supplement: Supplementary file 1 [file jcm-15-06792-s001.zip › Supplementary Material S6.pdf]

**Study excluded**	**Pooled OR**	**95% CI**	***p* value**	**I^2**	**τ^2**
Mazza et al.	1.76	1.13–2.74	0.012	0%	0.000
Bowen et al.	1.79	1.15–2.78	0.010	0%	0.000
Tamam et al.	1.65	1.06–2.58	0.027	0%	0.000
Lung et al.	1.80	1.16–2.81	0.009	0%	0.000
Orhan et al.	1.73	1.11–2.68	0.015	0%	0.000
Bayer-Topilsky et al.	2.01	1.15–3.52	0.014	0%	0.000
Beyazal et al.	1.84	1.03–3.29	0.041	0%	0.000
Yetkin et al.	1.78	1.14–2.77	0.011	0%	0.000
