# Supplementary material for: Mitral Valve Prolapse and Anxiety Disorders: A Systematic Review of Evidence in Both Directions and Meta-Analysis
Source: J Clin Med. 2026 Sep 1;15(17):6792. doi: 10.3390/jcm15176792 (PMC13566358; doi:10.3390/jcm15176792)
Supplement: Supplementary file 1 [file jcm-15-06792-s001.zip › Supplementary Material S7.pdf]

Study	Q1	Q2	Q3	Q4	Q5	Q6	Q7	Q8	Q9	Q10	Q11	Q12	Overall quality
Mazza D.L. [22]	Y	Y	N	N	Y	Y	NR	CD	N	Y	Y	N	**Fair**
Bowen R.C. [23]	Y	Y	N	Y	Y	Y	N	Y	N	Y	N	N	**Fair**
Tamam L. [24]	Y	Y	N	N	Y	Y	N	Y	N	Y	NR	N	**Fair**

Study	Q1	Q2	Q3	Q4	Q5	Q6	Q7	Q8	Q9	Q10	Q11	Q12	Q13	Q14	Overall quality
Lung F.W. [25]	Y	Y	NR	Y	NR	N	N	NA	CD	N	Y	NR	NA	N	**Fair**
Orhan A.L. [26]	Y	Y	NR	N	NR	N	N	Y	Y	N	Y	Y	NA	N	**Fair**
Dubey N.K. [27]	Y	Y	Y	Y	NR	N	N	NA	CD	N	CD	NA	NA	N	**Poor**
Bayer-Topilsky T. [28]	Y	Y	Y	N	Y	N	N	Y	Y	N	Y	NA	N	Y	**Good**
Çağlayan U. [29]	Y	Y	NR	Y	Y	N	N	Y	Y	N	N	NA	NA	N	**Fair**
Beyazal M. [30]	Y	Y	NR	Y	NR	N	N	Y	Y	N	Y	NR	NA	N	**Fair**
Yetkin Z.D. [31]	Y	Y	Y	Y	Y	N	N	Y	Y	N	Y	Y	NA	N	**Fair**
