# Supplementary material for: Mitral Valve Prolapse and Anxiety Disorders: A Systematic Review of Evidence in Both Directions and Meta-Analysis
Source: J Clin Med. 2026 Sep 1;15(17):6792. doi: 10.3390/jcm15176792 (PMC13566358; doi:10.3390/jcm15176792)
Supplement: Supplementary file 1 [file jcm-15-06792-s001.zip › Supplementary Material S8.pdf]

**Study**	**Q1**	**Q2**	**Q3**	**Q4**	**Q5**	**Q6**	**Q7**	**Q8**	**Q9**	**Q10**	**Q11**	**Q12**	**Overall quality**
Crowe et al. [32]	Y	Y	N	N	Y	Y	N	Y	N	Y	NR	N	**Fair**
Venkatesh et al. [33]	Y	Y	N	N	Y	Y	N	Y	N	Y	Y	N	**Fair**
Shear et al. [34]	Y	Y	N	CD	Y	Y	N	N	N	Y	Y	N	**Fair**
Carney et al. [36]	Y	Y	N	NA	Y	Y	N	Y	N	Y	Y	N	**Fair**
Toren et al. [37]	Y	Y	N	Y	Y	Y	N	Y	N	Y	Y	N	**Fair**
Filho et al. [38]	Y	Y	Y	CD	Y	Y	N	Y	N	Y	Y	Y	**Good**

**Study**	**Q1**	**Q2**	**Q3**	**Q4**	**Q5**	**Q6**	**Q7**	**Q8**	**Q9**	**Q10**	**Q11**	**Q12**	**Q13**	**Q14**	**Overall quality**
Liberthson et al. [35]	Y	Y	NR	Y	Y	N	N	NA	Y	N	Y	NR	NA	N	**Fair**
